# δ-Tocotrienol feeding modulates gene expression of EIF2, mTOR, protein ubiquitination through multiple-signaling pathways in chronic hepatitis C patients

**DOI:** 10.1186/s12944-018-0804-7

**Published:** 2018-07-21

**Authors:** Asaf A. Qureshi, Dilshad A. Khan, Shahida Mushtaq, Shui Qing Ye, Min Xiong, Nilofer Qureshi

**Affiliations:** 1 Department of Biomedical Science, School of Medicine, University of Missouri-Kansas City, 2411 Holmes Street, Kansas City, MO 64108 USA; 2 Department of Chemical Pathology and Endocrinology, Armed Forces Institute of Pathology (AFIP), National University of Medical Sciences, Rawalpindi, 64000 Pakistan; 3 Division of Experimental and Translational Genetics, Department of Pediatrics, Childern’s Mercy Hospital, 2401 Gillham Road, Kansas City, MO 64108 USA; 4 Department of Biomedical and Health Informatics, School of Medicine, University of Missouri-Kansas City, 2411 Holmes Street, Kansas City, MO 64108 USA; 5 Pharmacology/Toxicology, School of Pharmacy, University of Missouri-Kansas City, 2464 Charlotte Street, Kansas City, MO 64108 USA

**Keywords:** δ-Tocotrienol, Chronic hepatitis C, RNA-sequence, Gene expression of biomarkers, Causal network, Diseases and functions, Up-stream regulators, Canonical pathways

## Abstract

**Background:**

δ-Tocotrienol is a naturally occurring proteasome inhibitor, which has the capacity to inhibit proliferation and induce apoptosis in several cancer cells obtained from several organs of humans, and other cancer cell lines. Moreover, results of plasma total mRNAs after δ-tocotrienol feeding to hepatitis C patients revealed significant inhibition in the expression of pro-inflammatory cytokines (TNF-α, VCAM1, proteasome subunits) and induction in the expression of ICAM1 and IFN-γ after post-treatment. This down-regulation of proteasome subunits leads to autophagy, apoptosis of immune cells and several genes. The present study describes RNA-sequence analysis of plasma total mRNAs obtained from δ-tocotrienol treatment of hepatitis C patients on gene expression regulated by proteasome.

**Methods:**

Pooled specimens of plasma total mRNAs of pre-dose versus post-dose of δ-tocotrienol treatment of hepatitis C patients were submitted to RNA-sequence analyses. The data based on > 1 and 8-fold expression changes of 2136 genes were uploaded into “Ingenuity Pathway Analyses (IPA)” for core analysis, which describes possible canonical pathways, upstream regulators, diseases and functional metabolic networks.

**Results:**

The IPA of “molecules” indicated fold change in gene expression of 953 molecules, which covered several categories of biological biomarkers. Out of these, gene expression of 220 related to present study, 12 were up-regulated, and 208 down-regulated after δ-tocotrienol treatment. The gene expression of transcription regulators (ceramide synthase 3 and Mohawk homeobox) were up-regulated, and gene expression of 208 molecules were down-regulated, involved in several biological functions (HSP90AB1, PSMC3, CYB5R4, NDUFB1, CYP2R1, TNFRF1B, VEGFA, GPR65, PIAS1, SFPQ, GPS2, EIF3F, GTPBP8, EIF4A1, HSPA14, TLR8, TUSSC2). IPA of “causal network” indicated gene regulators (676), in which 76 down-regulated (26 s proteasomes, interleukin cytokines, and PPAR-ligand-PPA-Retinoic acid-RXRα, PPARγ-ligand-PPARγ-Retinoic acid-RARα, IL-21, IL-23) with significant *P*-values. The IPA of “diseases and functions” regulators (85) were involved with cAMP, STAT2, 26S proteasome, CSF1, IFNγ, LDL, TGFA, and microRNA-155-5p, miR-223, miR-21-5p. The IPA of “upstream analysis” (934) showed 57 up-regulated (mainly 38 microRNAs) and 64 gene regulators were down-regulated (IL-2, IL-5, IL-6, IL-12, IL-13, IL-15, IL-17, IL-18, IL-21, IL-24, IL-27, IL-32), interferon β-1a, interferon γ, TNF-α, STAT2, NOX1, prostaglandin J2, NF-κB, 1κB, TCF3, and also miRNA-15, miRNA-124, miRNA-218-5P with significant activation of Z-Score (*P* < 0.05).

**Conclusions:**

This is first report describing RNA-sequence analysis of δ-tocotrienol treated plasma total mRNAs obtained from chronic hepatitis C patients, that acts via multiple-signaling pathways without any side-effects. These studies may lead to development of novel classes of drugs for treatment of chronic hepatitis C patients.

**Electronic supplementary material:**

The online version of this article (10.1186/s12944-018-0804-7) contains supplementary material, which is available to authorized users.

## Background

**Fig. 1:**
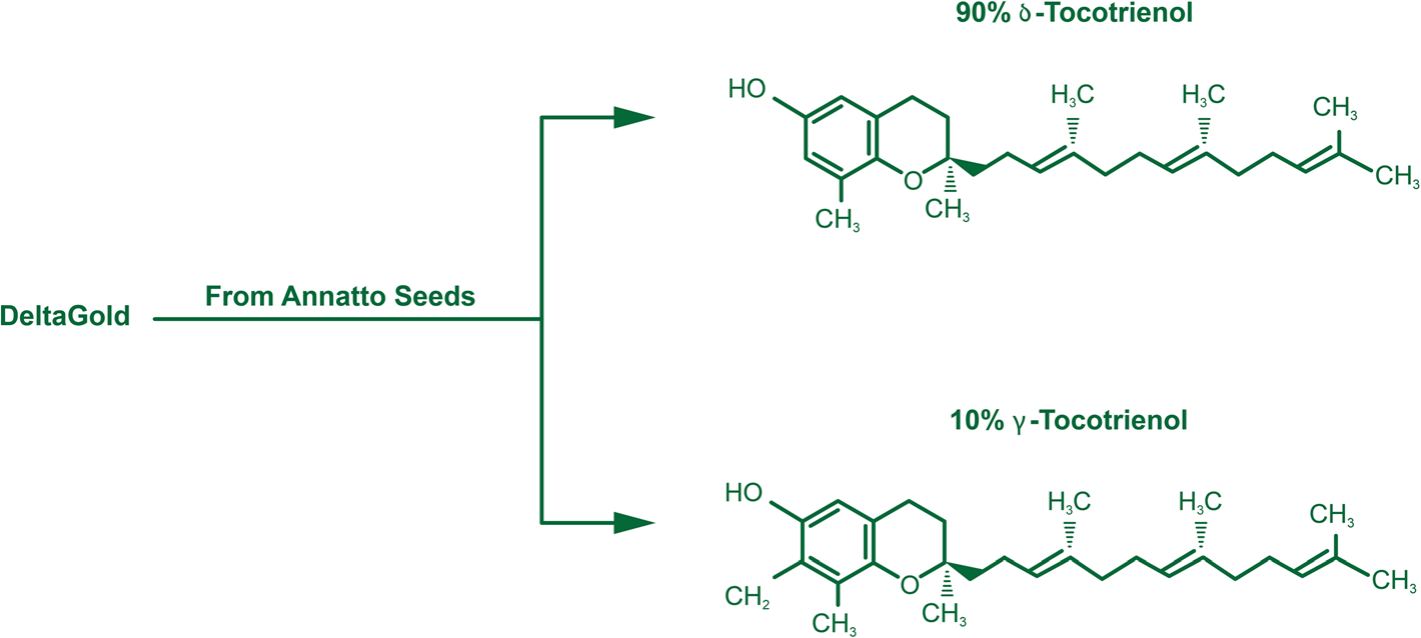
Chemical structure of δ-tocotrienol (similar figure was published in our publication-54. Qureshi et al., Journal of Clinical & Experimental Cardiology. 2015;6:4. 10.4172/2155-9880.1000367 [54]

We have recently reported that δ-tocotrienol is a potent anti-cancer agent (liver, pancreas, prostrate, breast cancer cell lines, Hela, melanoma, B lymphocytes and T-cells), and also a modulator of proteasome function, as compared to other outstanding proteasome inhibitors (thiostrepton, 2-methoxyestradiol, and quercetin) [1]. Moreover, plasma total mRNAs obtained from δ-tocotrienol treated hepatitis C patients showed significant inhibition in the expression of pro-inflammatory cytokines (TNF-α and VCAM-1), and induction in expression of ICAM-1, IFN-γ, whereas proteasome subunits X, Y, Z, LMP7, LMP2, LMP10 (22–44%) were significantly inhibited compared to pre-dose values, and this down-regulation of proteasome subunits leads to autophagy and apoptosis of cells [1]. The present study is an extension of these findings to study the effect of δ-tocotrienol (Fig. 1) treatment of chronic hepatitis C patients in their plasma mRNAs using RNA-Sequencing by Ingenuity Pathway Analysis (IPA). The viral infection with hepatitis C is responsible for a vast majority of chronic hepatitis cases over 180 million people worldwide, which is further supported by epidemiological and clinical studies have also demonstrated a causative role of viral infection of hepatitis C in the development of hepatocellular carcinoma [2]. These figures are alarming, as patients currently asymptomatic with relatively mild disease may eventually progress to complications of chronic liver diseases, like cirrhosis, and hepatocellular carcinoma [3]. The mechanisms of liver disease are not fully understood.

The mechanisms that contribute to the pathogenesis of hepatitis virus-related liver infections are diverse and very complex. Investigation of altered cellular mechanisms through gene profiling techniques has improved the clear understanding of various disease processes and development of novel therapeutic targets [4]. Earlier, techniques applied for studying gene expression profiling included microarrays, which analyzes quantitative expression of thousands of genes, and time consuming real-time PCR assays that gives only small number of expression of genes. These tools have been used previously for identification of differentially expressed genes in hepatitis C virus associated cirrhosis and carcinoma [5]. In summary, these changes in gene expression were associated with immune response, fibrosis, cellular growth, proliferation, and apoptosis [5–7]. Nowadays, similar estimation carried out by RNA-sequence procedure, which will provide very accurate gene expression of several virus important biological functions and biomarkers.

The genotype hepatitis C is an important determinant of the response to treatment, and differences found in clinical outcomes of the disease with respect to infection of various genotypes [6–8]. The genotype 3 is the most prevalent genotype around the world compared to other genotype infection [8]. In the present study we will identify altered cellular processes in chronic hepatitis C patients after treatment with δ-tocotrienols. The main purpose of this preliminary study was to isolate plasma total mRNAs from a few participants after δ-tocotrienol treatment of chronic hepatitis C patients, and to carry out RNA-sequence analysis, which quantified mRNA expression of a large number of genes in pooled specimens of pre-dose versus post-dose of δ-tocotrienol treatment of chronic hepatitis C patients. The gene expression data was analyzed by “Ingenuity Pathway Analysis”, which would reveal the cellular and biological mechanisms at the molecular level in plasma total mRNAs obtained from chronic hepatitis C patients.

## Methods

### Materials

DeltaGold 125 mg softgels from annatto seeds (typical composition 90% δ-tocotrienol and 10% γ-tocotrienol) were supplied by American River Nutrition, Inc. (Hadley, MA, USA). RNeasy mini kit was obtained from QIAGEN Sciences (Germantown, MD, USA).

### Impact of δ-tocotrienol in chronic hepatitis C patients

The study was carried out in Pakistan Ordinance Factory (POF) Hospital, Wah Cantonment, Rawalpindi, Pakistan; in collaboration with department of biomedical Sciences, University of Missouri-Kansas City, MO, USA. The study protocol was registered (IRB # 129–2015) was approved by Institutional Review Board of POF, Rawalpindi, Pakistan. The study was carried out under a FDA approved IND number 36906**.** The hepatitis C antibody test was purchased from Sigma Chemical Co., St. Louis, USA. The second diagnosing hepatitis C test is RNA PCR test was obtained from the EDTA treated fresh whole blood by using total RNA purification kit # 17200 (NORGEN Bioteck Corporation, Thorold, ON, Canada).

### RNA-Sequence Analyses of plasma total RNAs obtained from EDTA treated whole blood after feeding δ-tocotrienol for 6-weeks to hepatitis C patients

The details of study design, inclusion/exclusion criteria, experimental design, and physical characteristics of hepatitis C patients were same as reported [1]. In short, the total mRNA was extracted from plasma of EDTA treated fresh whole blood of each hepatitis C patients (*n* = 14) fed δ-tocotrienol (500 mg/d) for 6 weeks by total RNA purification kit (NORGEN Bioteck Corporation, Thorold, ON, Canada). The purity of total RNAs (stored − 80 °C) was estimated by the ratios of 260/280 (2.02–2.08) of all samples, which was determined using Thermo Scientific NanoDrop 1000 Spectrophotometer. The mRNAs samples from Pakistan were brought in person (by Dr. Dilshad A. Khan in dry ice to avoid any degradation of RNAs) to UMKC, Medical School after approval by (Compliance officer Mr. Christopher Winders, and Chemical/Biological Safety officer Mr. Mike Philips) members of University of Missouri Kansas City institutional review board.

The results of most important cytokines and other biomarkers associated with the present investigation were estimated by real-time RT-PCR by using plasma total RNAs purified from pre-dose versus post-dose samples after feeding δ-tocotrienol for 6-weeks to chronic hepatitis C patients has been published recently [1], therefore present manuscript lacks in vitro estimations of RT-PCR data. The same plasma total RNAs were used in the present study.

The RNA-Sequence analyses were carried out at Division of Experimental and Translational Genetics, Children’s Mercy Hospital, Kansas City, MO. Five randomized samples selected of total RNAs of hepatitis C patients, and combined. Total mRNAs of combined samples were purified by Biostic Blood Total RNA Isolation Kit (MOBIO Laboratories, Inc). The purified total mRNAs were further purified and concentrated to 10.0 μl by using by Gene Jet RNA Clean up and Concentration Micro Kit (Thermo Scientific, EU, Lithuania). The purity of these RNAs was further determined in the Division of Experimental and Translational Genetic & Core of Omic Research (The Children Mercy Hospital, Kansas City, MO) by their own instruments for quality control and quantity of each sample to make sure that each sample is up to standard before putting into a NGS run. The concentrated total mRNAs of each set was converted to cDNA, and total RNA-Seq carried out. Gene expression level and fold change (post vs pre-dose) of FPKM were calculated at > 1, > 2, or > 5 levels at 2-fold, 4-fold, and 8-fold after filtering several million fold up-regulated and down-regulated genes (Table 1).

**Table 1:** Estimation of basic RNA-sequence expresion unit (FPKM) of δ-tocotrienol treated hepatitis C patients^1^

#	RNA-Seq expression unit	Number of genes	Genes based on 2-fold	Genes based on 4-fold	Genes based on 8-fold
1	FPKM > 1	12614	9480	5369	2136
2	FPKM > 2	7426	1366	696	527
3	FPKM > 5	3323	379	285	268

^1^The gene expression level and fold change (post-dose vs pre-dose) of FPKM were calculated at more than 1, 2, or 5 at 2-fold, 4-fold, and 8-fold after filtering million-fold up-regulation and down-regulation. The RNA-seq analyses data based on FPKM >1 and 8-fold change of 2136 genes (0 values were replaced with 0.001) of ratios of post-dose over pre-dose treatment of δ-tocotrienol to hepatitis C patients was submitted into “Ingenuity Pathway Analyses (IPA)” for core analysis (Ingenuity Systems, Redwood City, CA)

### Statistical analyses

These data were analyzed by IPA program of treatment-mediated effects as post-dose versus pre-dose. The statistical significance level was set at 5% (*P* < 0.05).

## Results

### Genome-wide profiling experiment of plasma mRNAs obtained from pre-dose and post-dose δ-tocotrienol treatment of hepatitis C patients

The RNA-Sequence analysis was based on FPKM > 1 and 8-fold change of 2136 genes (0 values replaced with 0.001; Table 1) ratios of post-dose over pre-dose treatment of δ-tocotrienol to hepatitis C patients were uploaded into “Ingenuity Pathway Analyses (IPA)” for core analysis (Ingenuity Systems, Redwood City, CΑ). The various genes associated with different biological functions and biomarkers are from “Ingenuity Knowledge Base” generated molecular networks, according to biological as well as molecular functions. These include canonical pathways, upstream regulatory analysis, and disease-based functional network, which helped discovering the list of several biomarkers. The core analysis was carried out with the settings of indirect and direct relationship between focused molecules based on experimentally observed data and human databases in the “Ingenuity Knowledge Base” were considered as the data sources in these analyses and pathways.

### “Molecules” affected by δ-tocotrienol feeding to hepatitis C patients

**Fig. 2:**
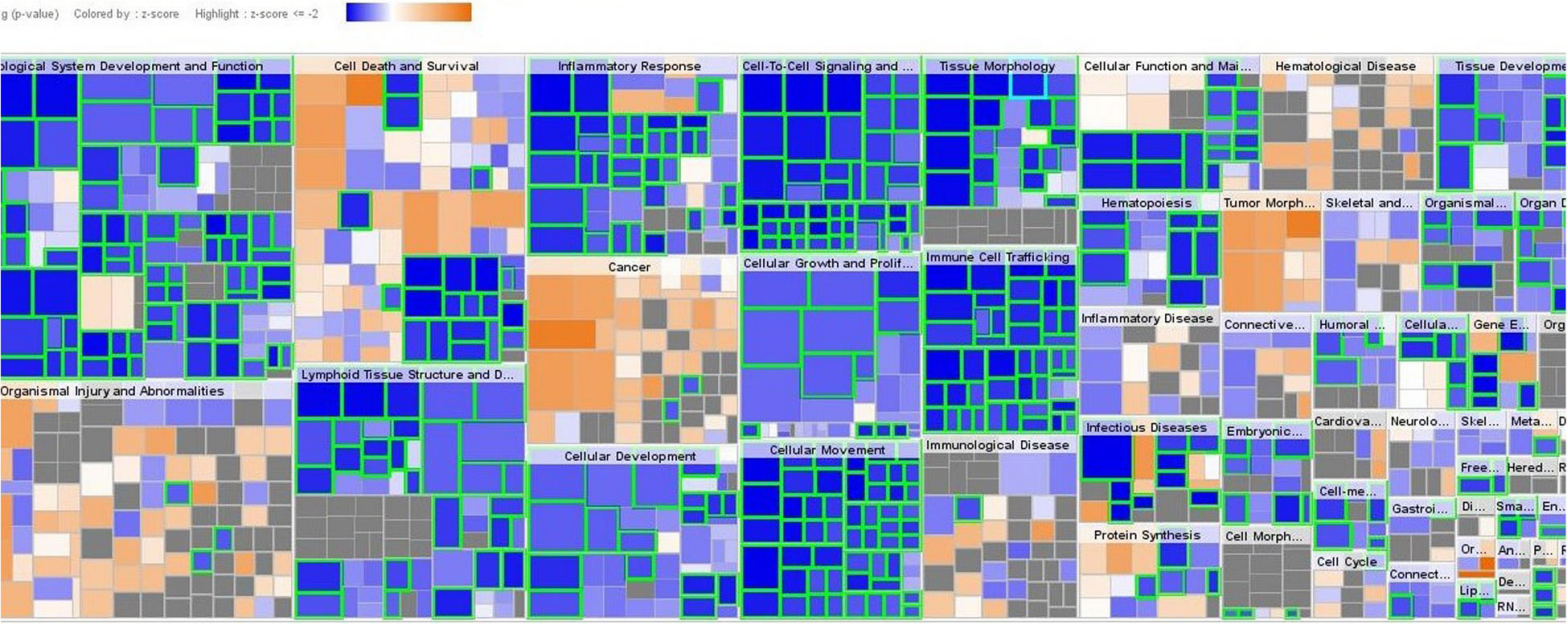
Effect of several biological biomarkers in “diseases and functions” of heat map in plasma of total mRNAs obtained from δ-tocotrienol treatment of hepatitis C patients. The fold change expression of several biological functions (hematological system, function development, cell death, survival, inflammatory response, cell to cell signaling, cancer, organism injury, organism abnormalities, cellular development and immunological diseases) are illustrated in heat map

The IPA of “molecules section” indicates fold changes in gene expression of 953 genes, which covered several categories of biological biomarkers, which are presented in the heat-map of this section (Fig. 2). Out of these, expression of 220 genes were related to present study, and only 12 genes were up-regulated (Table 2), and remaining 208 genes of various biomarkers were down-regulated after δ-tocotrienol treatment (Table 3). The ceramide synthase 3 and Mohawk homeobox were only two up-regulated genes involved as transcription regulators. The down-regulated gene expression of 208 molecules are involved in several biological functions (Additional file 1: Table S1, Additional file 2: Table S2 and Additional file 3: Table S3). The functions of these regulators are ATPase NA^+^/K^+^ transporting subunit α1, apolipoprotein B, proteasome 26S subunits, NADH ubiquinone oxidoreductase subunits B1, B9, cytochrome b5 reductase 4, autophagy related 4 ~ 5, cytochrome P450 family, TNF receptor superfamily 1B, RAS P21 protein activator 2, ubiquitin conjugating enzyme B2 J1, several other types of ubiquitin proteasome subunits, and protein inhibitor of activated STAT1 (Table 3). Similarly, gene regulator of G-protein signaling 2, nuclear factor of activated T-cells 2 interacting protein, TNF-α induced protein 8, C-X-C motif chemokines ligand 1, RNA polymerase II subunit H, tumor suppressor candidate 2, splicing factor 3b subunit 5, and several miRNAs (877, 1250,140), RNAs, tRNAs are reported in Table 3. The summary of most important down-regulated biomarkers are HSP90AB1, IL-16, autophagy, TNFSF1B, VEGFA, NFIL3, UBP1, USP25, RASA3, USP15, UBE4A, USP19, PSMG3, IL-27RA, SCP2, IFNGR1, ID2, TUSC2, IL-1R2, IL18RP, IRF2, PCNA1250,77,40 and several tRNAs (Table 3).

**Table 2:** Effect of δ-tocotrienol on up-regulation of fold change gene expression of “Molecules” section (12) of IPA analysis in hepatitis C patients

Up-regulation				
#	Symbol	Entrez Gene Name	Expr Fold Change	Type(s)
1	HIST1H2AD	histone cluster 1 H2A family member d	1804955.068	other
2	HHIPL2	HHIP like 2	28.710	other
3	RPP38	ribonuclease P/MRP subunit p38	24.946	enzyme
4	CERS3	ceramide synthase 3	19.082	transcription regulator
5	HBG1	hemoglobin subunit gamma 1	17.945	other
6	MT-TQ	tRNA	14.252	other
7	AKR1D1	aldo-keto reductase family 1 member D1	14.056	enzyme
8	TSPAN15	tetraspanin 15	11.523	other
9	HBG2	hemoglobin subunit gamma 2	11.413	other
10	MKX	mohawk homeobox	9.573	transcription regulator
12	P4HA3	prolyl 4-hydroxylase subunit alpha 3	8.686	enzyme

**Table 3:** Effect of δ-tocotrienol on down-regulation of fold change gene expression of “Molecules” section (64) of IPA analysis in hepatitis C patients

Down-regulation				
#	Symbol	Entrez Gene Name	Expr Fold Change	Type(s)
1	ATP1A1	ATPase Na+/K+ transporting subunit alpha 1	-8.014	transporter
2	HSP90AB1	heat shock protein 90 alpha family class B member 1	-8.049	enzyme
3	APOBEC3A	apolipoprotein B mRNA editing enzyme catalytic subunit 3A	-8.163	enzyme
4	CXCR2	C-X-C motif chemokine receptor 2	-8.208	G-protein coupled receptor
5	IL16	interleukin 16	-8.239	cytokine
6	PSMC3	proteasome 26S subunit, ATPase 3	-8.346	transcription regulator
7	NDUFB9	NADH:ubiquinone oxidoreductase subunit B9	-8.354	enzyme
8	CYB5R4	cytochrome b5 reductase 4	-8.367	enzyme
9	ATG3	autophagy related 3	-8.376	enzyme
10	CREB1	cAMP responsive element binding protein 1	-8.452	transcription regulator
12	NDUFB1	NADH:ubiquinone oxidoreductase subunit B1	-8.566	enzyme
13	PDE3B	phosphodiesterase 3B	-8.568	enzyme
14	IGF2R	insulin like growth factor 2 receptor	-8.68	transmembrane receptor
15	CYP2R1	cytochrome P450 family 2 subfamily R member 1	-8.682	enzyme
16	NDUFA11	NADH:ubiquinone oxidoreductase subunit A11	-8.686	enzyme
17	IGSF6	immunoglobulin superfamily member 6	-8.712	transmembrane receptor
18	TNFRSF1B	TNF receptor superfamily member 1B	-8.746	transmembrane receptor
19	PRPF18	pre-mRNA processing factor 18	-8.777	transporter
20	SERP1	stress associated endoplasmic reticulum protein 1	-8.872	other
21	UBE2J1	ubiquitin conjugating enzyme E2 J1	-8.874	enzyme
22	VEGFA	vascular endothelial growth factor A	-8.933	growth factor
23	GYS1	glycogen synthase 1	-9.027	enzyme
24	GPR65	G protein-coupled receptor 65	-9.054	G-protein coupled receptor
25	ILF2	interleukin enhancer binding factor 2	-9.105	transcription regulator
26	OSBPL11	oxysterol binding protein like 11	-9.201	other
27	PSMA5	proteasome subunit alpha 5	-9.31	peptidase
28	PIAS1	protein inhibitor of activated STAT 1	-9.326	transcription regulator
29	TRAF7	TNF receptor associated factor 7	-9.341	enzyme
30	COX14	COX14, cytochrome c oxidase assembly factor	-9.447	other
31	RPS26	ribosomal protein S26	-9.456	other
32	SFPQ	splicing factor proline and glutamine rich	-9.469	other
33	ATF4	activating transcription factor 4	-9.515	transcription regulator
34	PECAM1	platelet and endothelial cell adhesion molecule 1	-9.552	other
35	GPS2	G protein pathway suppressor 2	-9.56	transcription regulator
36	NFIL3	nuclear factor, interleukin 3 regulated	-9.568	transcription regulator
37	PSMB8	proteasome subunit beta 8	-9.709	peptidase
38	UBP1	upstream binding protein 1 (LBP-1a)	-9.718	transcription regulator
39	RAP2C	RAP2C, member of RAS oncogene family	-9.792	enzyme
40	PIBF1	progesterone immunomodulatory binding factor 1	-9.876	other
41	USP25	ubiquitin specific peptidase 25	-9.911	peptidase
42	FRS2	fibroblast growth factor receptor substrate 2	-9.962	kinase
43	PSMB4	proteasome subunit beta 4	-10.119	peptidase
44	USP15	ubiquitin specific peptidase 15	-10.16	peptidase
45	UBA52	ubiquitin A-52 residue ribosomal protein fusion product 1	-10.176	enzyme
46	UBE4A	ubiquitination factor E4A	-10.189	enzyme
47	GTPBP8	GTP binding protein 8 (putative)	-10.19	other
48	USP19	ubiquitin specific peptidase 19	-10.713	peptidase
49	TNFAIP8	TNF alpha induced protein 8	-10.974	other
50	HSPA14	heat shock protein family A (Hsp70) member 14	-10.978	peptidase
51	TLR8	toll like receptor 8	-11.975	transmembrane receptor
52	IL27RA	interleukin 27 receptor subunit alpha	-12.004	transmembrane receptor
53	SCP2	sterol carrier protein 2	-13.672	transporter
54	IFNGR2	interferon gamma receptor 2	-13.844	transmembrane receptor
55	ID2	inhibitor of DNA binding 2, HLH protein	-14.133	transcription regulator
56	TUSC2	tumor suppressor candidate 2	-15.922	other
57	IL2RG	interleukin 2 receptor subunit gamma	-16.787	transmembrane receptor
58	IL1R2	interleukin 1 receptor type 2	-19.547	transmembrane receptor
59	IRF2	interferon regulatory factor 2	-22.655	transcription regulator
60	PTGS2	prostaglandin-endoperoxide synthase 2	-25.841	enzyme
61	mir-877	microRNA 877	-4497.07	microRNA
62	mir-1250	microRNA 1250	-4755.79	microRNA
63	mir-140	microRNA 140	-5668.259	microRNA
64	KLRC4-KLRK1/KLRK1	killer cell lectin like receptor K1	-1565687.642	transmembrane receptor

### “Causal Networks” affected by δ-tocotrienol feeding to hepatitis C patients

The down-regulation of several biomarkers of “causal network” of IPA of RNA samples obtained after treatment with δ-tocotrienol of chronic hepatitis C patients is described in Tables 4 and 5.

**Table 4:** Effect of δ-tocotrienol on up-regulation (24) of fold change gene expression in "causal netwworks" section of IPA analysis in hepatitis C patients

#	Master Regulator	Molecule Type	Part. regulators^1^	Depth	Pred Acti State^2^	Act. Z-Score^3^	*P*-Value Over^4^	Network Bi-Corr^5^	Causal Net^6^	Target-Con-Re^7^
A	Up-regulation									
1	leuprolide	biologic drug	26s Proteasome,AKT1	3	Activated	2.104	8.5E-10	0.0032	217 (71)	69
2	HLA-DR	complex	26s Proteasome,AR,ATR	3	Activated	5.458	3.44E-09	0.0145	260 (87)	86
3	PRDX1	enzyme	26s Proteasome,ABL1	3	Activated	7.084	1.73E-08	0.0427	250 (76)	75
4	alefacept	bilologic drug	alefacept, AP1,CD2	3	Activated	2.278	2.50E-07	0.0222	85 (20)	20
5	juglone	chemical toxicant	CASP3,FOS,juglone,JUN	2	Activated	2.449	0.00000682	0.0272	54 (9)	9
6	mir-148	microRNA	mir-148	1	Activated	2.000	0.00103	0.0055	4 (1)	1
7	26s Proteasome	complex	26s Proteasome	1	Activated	2.840	0.00167	0.0476	15 (1)	1
8	mir-122	microRNA	mir-122	1	Activated	3.317	0.00189	0.022	11 (1)	1
9	mir-19	microRNA	mir-19	1	Activated	2.236	0.002	0.0185	5 (1)	1
10	mir-9	microRNA	mir-9	1	Activated	2.000	0.00473	0.0203	4 (1)	1
11	IL2RG	transmembrane	IL2RG	1	Activated	0.000	0.00181	0.0188	8 (1)	1
12	miR-2682-5p (other miRNAs w/seed AGGC)	mature microRNA	miR-2682-5p (miRNAs)	1	Activated	1.414	0.00584	0.0073	2 (1)	1
13	alpha-tocopherol succinate	chemical drug	alpha-tocopherol succinate	1	Activated	0.000	0.00597	0.0316	4 (1)	1
14	mir-199	microRNA	mir-199	1	Activated	1.732	0.00849	0.0258	3 (1)	1
15	mir-138	microRNA	mir-138	1	Activated	1.414	0.0113	0.0239	2 (1)	1
16	miR-330-5p (other miRNAs w/seed CUCU)	mature microRNA	miR-330-5p (and other	1	Activated	1.414	0.0113	0.0209	2 (1)	1
17	mir-326	microRNA	mir-326	1	Activated	1.414	0.0113	0.0191	2 (1)	1
18	mir-32	microRNA	mir-32	1	Activated	1.414	0.0113	0.0304	2 (1)	1
19	LAMP2	enzyme	LAMP2	1	Activated	0.000	0.0113	0.0251	2 (1)	1
20	mir-218	microRNA	mir-218	1	Activated	1.732	0.0183	0.0398	3 (1)	1
21	UBA7	enzyme	UBA7	1	Activated	1.414	0.0183	0.0416	2 (1)	1
22	miR-147a (miRNAs w/seed UGUGUGG)	mature microRNA	miR-147a (other miRNAs)	1	Activated	1.000	0.0448	0.0417	1 (1)	1
23	miR-504-5p (other miRNAs w/seed GACC)	mature microRNA	miR-504-5p (miRNAs)	1	Activated	1.000	0.0448	0.0417	1 (1)	1
24	BI 2536	chemical drug	26s Proteasome,ABL1	3	Activated	1.331	2.06E-12	0.0034	249 (50)	49

^1^Part. Regulators = Paticipating Regulators; ^2^Pred Acti state = Predicted Acitivation State; ^3^Act. Z-Score = Activation Z-Score; ^4^*P*-Value Over. = *P*-Value Overlap; ^5^Network Bi-Corr = Network Bias-Corrected *P*-Values; ^7^Target-Con-Re. = Target Connected regulators

**Table 5:** Effect of δ-tocotrienol on down-regulation (74) of fold change gene expression in "causal netwworks" section of IPA analysis in hepatitis C patients

#	Master Regulator	Molecule Type	Part. regulators^1^	Depth	Pred Acti State^2^	Act. Z-Score^3^	*P*-Value Over^4^	Network Bi-Corr^5^	Causal Net^6^	Target-Con-Re^7^
B	Down-regulation									
25	JAK1/2	group	26s Proteasome,Akt,AKT1	3	Inhibited	-7.511	2.54E-14	0.0008	295 (81)	80
26	PPAR ligand-PPAR-Retinoic acid-RXRα	complex	26s Proteasome,Akt,AKT1	3	Inhibited	-4.459	3.31E-13	0.0131	306 (61)	60
27	LXR ligand-LXR-Retinoic acid-RXRα	complex	26s Proteasome,Akt,AR	3	Inhibited	-4.815	4.17E-13	0.0085	290 (58)	57
28	PPARγ ligand-PPARγ-Retinoic acid-RARα	complex	26s Proteasome,Akt,AKT1	3	Inhibited	-4.230	4.23E-13	0.0121	306 (66)	65
29	PXR ligand-PXR-Retinoic acid-RXRα	complex	26s Proteasome,AKT1	3	Inhibited	-4.432	3.33E-12	0.0221	294 (58)	58
30	RAR ligand-RARα-Retinoic acid-RXRα	complex	26s Proteasome,Akt,AKT1	3	Inhibited	-5.396	3.52E-12	0.039	297 (57)	56
31	Vegf Receptor	group	26s Proteasome,ABL1,Akt	3	Inhibited	-5.056	1.56E-11	0.0052	276 (93)	90
32	FXR ligand-FXR-Retinoic acid-RXRα	complex	26s Proteasome,Akt,AKT1	3	Inhibited	-5.100	1.96E-11	0.0484	291 (56)	55
33	hydrogen sulfide	chemical - endogenous mammalian	26s Proteasome,Akt,AKT1	3	Inhibited	-4.222	2.15E-11	0.0013	237 (92)	89
34	NLK	kinase	26s Proteasome,AKT1,Alp	3	Inhibited	-3.429	8.72E-11	0.0375	248 (50)	45
35	CD80	transmembrane receptor	CD28,CD80,IFNG,IL4	2	Inhibited	-6.267	1.32E-10	0.003	132 (8)	8
36	Pdgfra-Pdgfrb	complex	26s Proteasome,AKT1,AR	3	Inhibited	-7.878	1.37E-10	0.0184	285 (93)	89
37	Klra7 (includes others)	transmembrane receptor	26s Proteasome,Akt,AR	3	Inhibited	-7.445	1.44E-10	0.0324	291 (93)	93
38	FLT4	transmembrane receptor	26s Proteasome,Akt,AR	3	Inhibited	-5.020	1.46E-10	0.0177	280 (80)	78
39	Vegfr dimer	complex	26s Proteasome,AKT1,AR	3	Inhibited	-7.071	1.59E-10	0.0178	242 (61)	58
40	lipopolysaccharide	chemical drug	lipopolysaccharide	1	Inhibited	-7.668	2.75E-10	0.0045	120 (1)	1
41	TEK	kinase	26s Proteasome,ADRB2	3	Inhibited	-4.954	3E-10	0.0124	274 (93)	93
42	LATS1	kinase	26s Proteasome,ARID4A	3	Activated	4.680	3.43E-10	0.0322	250 (56)	54
43	NYAP1	other	26s Proteasome,Akt,AKT1	3	Inhibited	-6.264	3.54E-10	0.0304	281 (86)	85
44	MYO16	other	26s Proteasome,Akt,AKT1	3	Inhibited	-6.264	3.54E-10	0.0304	281 (86)	85
45	NYAP2	other	26s Proteasome,Akt,AKT1	3	Inhibited	-6.264	3.54E-10	0.0304	281 (86)	85
46	IRS	group	26s Proteasome,ADRB2	3	Inhibited	-5.548	1.63E-09	0.0456	269 (77)	74
47	FAK-Src	complex	26s Proteasome,ABL1,Akt	3	Inhibited	-6.839	2.41E-09	0.043	273 (90)	86
48	Plk	group	26s Proteasome,Akt,AKT1	3	Inhibited	-2.500	2.77E-09	0.0425	219 (55)	50
49	G-protein beta	group	26s Proteasome,ADORA2A	3	Inhibited	-5.647	3.22E-09	0.0309	283 (103)	99
50	ADRA1B	G-protein coupled receptor	26s Proteasome,ADRA1B	3	Inhibited	-6.238	4.49E-09	0.0406	278 (86)	85
51	IL2	cytokine	IL2	1	Inhibited	-4.619	8.23E-09	0.0004	48 (1)	1
52	propolis	biologic drug	26s Proteasome,AKT1	3	Inhibited	-2.829	1.78E-08	0.0482	231 (76)	73
53	exenatide	biologic drug	26s Proteasome,Akt,AMPK	3		-1.432	2.36E-08	0.0088	236 (88)	88
54	imidazole	chemical - endogenous mammalian	26s Proteasome,ADORA2A	3		1.091	2.79E-08	0.05	243 (75)	70
55	LETM1	other	Akt,AMPK,APP,AR	3		-1.023	0.000000069	0.036	215 (64)	63
56	IL-2R	complex	IL-2R,IL2RA,IL2RG,JAK1	2	Inhibited	-3.491	0.00000012	0.0103	84 (14)	13
57	IL23	complex	IL12B,IL23,JAK2,MTOR	2	Inhibited	-7.155	0.000000165	0.0112	80 (9)	9
58	IL15	cytokine	IL15	1	Inhibited	-2.121	0.000000551	0.0009	32 (1)	1
59	TH17 Cytokine	group	IL17A,IL21,IL22,TH17	2	Inhibited	-4.323	0.000000813	0.0037	39 (4)	4
60	IL4R	transmembrane receptor	IL4,IL4R,IRS1,IRS2,JAK	2	Inhibited	-4.503	0.00000102	0.0252	75 (13)	12
61	IL21	cytokine	IL21	1	Inhibited	-2.985	0.00000527	0.0028	22 (1)	1
62	SATB1	transcription regulator	SATB1	1		1.528	0.00000669	0.0011	21 (1)	1
63	cyclosporin A	biologic drug	cyclosporin A	1		1.441	0.0000108	0.0163	39 (1)	1
64	IL12RB2	transmembrane receptor	IL12 (family),IL12RB2	2	Inhibited	-4.116	0.0000233	0.0103	34 (4)	3
65	mir-26	microRNA	Akt,mir-26	2		0.192	0.0000247	0.0126	27 (2)	2
66	mir-221	microRNA	Akt,mir-221	2		-0.192	0.0000247	0.0129	27 (2)	2
67	IL5	cytokine	IL5	1	Inhibited	-4.914	0.0000541	0.0136	28 (1)	1
68	ropivacaine	chemical drug	Akt,NOS3,Pkc(s)	2		-1.029	0.0000544	0.0289	34 (5)	4
69	UCP3	transporter	IRS1,IRS2,PI3K	2		-1.961	0.0000657	0.0231	26 (4)	3
70	AIF1	other	AIF1,Akt,BAD	2		-1.177	0.0000657	0.0211	26 (3)	3
71	IFN Beta	group	IFN Beta	1	Inhibited	-2.138	0.00082	0.043	14 (1)	1
72	PDGFD	growth factor	PDGFD	1		-0.577	0.000838	0.0044	3 (1)	1
73	PARP9	enzyme	PARP9	1	Inhibited	-2.236	0.00123	0.0073	5 (1)	1
74	PPP1R14B	phosphatase	PPP1R14B	1		-1.732	0.00162	0.005	3 (1)	1

^1^Part. Regulators = Paticipating Regulators; ^2^Pred Acti state = Predicted Acitivation State; ^3^Act. Z-Score = Activation Z-Score; ^4^*P*-Value Over. = *P*-Value Overlap; ^5^Network Bi-Corr = Network Bias-Corrected *P*-Values; ^7^Target-Con-Re. = Target Connected regulators

There were 676 gene regulators identified in this section, and only 98 regulators were associated with present study, indicating significant *P*-values for all regulators (Tables 4 and 5). The fold change gene expression of 24 was up-regulated (Table 4) and 74 down-regulated (Table 5). This section includes down-regulated gene expression of 26S proteasomes, interleukin cytokines, and PPAR-ligand-PPA-Retinoic acid-RXTα, PPARγ-ligand-PPARγ-Retinoic acid-RARα, IL-7R, CD80, IRS, IL-2, IL-2RG, IL-5, IL-15, IL-21, IL-23 and several types of microRNAs (miRNAs) as shown in Table 5. The activation Z-Score, *P*-values, network bias-corrected and causal network values were in descending order of all these gene biomarkers (Tables 4 and 5).

### “Diseases and functions” affected by δ-tocotrienol feeding to hepatitis C patients

The IPA of RNAs obtained from effect of δ-tocotrienol treatment of chronic hepatitis C patients on relative percentage relationship of gene regulators (70) of “diseases and functions” reported in Table 6. In this section, percentage relationships of main regulators were AP1, cAMP, EIF2AK2 2RL1, IL-17A, IL-1RN, KITLG, miRNA-155-5p, STAT2 (48%; 43/90), 26S proteasome, CSF1, IFNG, IL-17A, IRF4, LDL, RELA, TGFA (43%; 17/40); mir-223 (0%; 0/2), IL-15 (100%; 1/1), IL-1Β (0%; 0/1), and miR-21-5p (100%; 1/1) (Table 6). The consistency score of these regulators varied from 1.73 ~ 36.34, total regulars (1–9), total node (5–57), diseases and functions total varied 1–10 as shown in Table 6.

**Table 6:** Effects of δ-tocotrienol treatment on "Regulator Effects" section (70) of IPA analysis of "Diseases and Functions" in hepatitis C patients

ID	Consistency	Node	Regulator	Regulators	Target	Disease &	Diseases & Functions	Known Regulator-Disease/
	Score	Total	Total		Total	Fuunctions Totals		Function Relationship
1	36.338	57	9	Ap1,CAMP,EIF2AK2,IL17A,IL1R,miR-155-5,STAT2	38	10	activation of phagocytes	48% (43/90)
2	32.199	69	13	26s Proteasome,ANGPT2,Ap1,BCL2,CAMP,CEBPA,TGFA	45	11	activation of antigen presenting cells	40% (57/143)
3	30.414	57	12	26s Proteasome,CAMP,CSF1,F2RL1,IL17A,miR-21-5p,TGFA	37	8	activation of myeloid cells	32% (31/96)
4	30.375	97	13	Ap1,CAMP,CCL5,EIF2AK2,F2RL1,FGF10,IL17A,	64	20	accumulation of l cells,leukopoiesis	38% (99/260)
5	28.605	56	10	26s Proteasome,BCL2,CAMP,STAT3,TGFA,TGM2	37	9	adhesion of blood cells	36% (32/90)
6	25.456	49	8	26s Proteasome,F2RL1,IL1RN,IRF4,KLF3,STAT3,TGFA,	32	9	adhesion of immune cells	26% (19/72)
7	25.126	127	20	ANGPT2,Ap1,CAMP,CST5,ETS1,F2RL1,IFNL1,IGF1,IL17A,	92	15	cell movement of granulocytes	40% (121/300)
8	24.82	53	8	26s Proteasome,BCL2,CSF1,F2RL1,IL1RN,STAT3,TGFA,	38	7	adhesion of blood cells	41% (23/56)
9	23.333	50	7	CAMP,F2RL1,IL17A,mir-10,NRG1,TGFA,Tlr	36	7	cell viability of tumor cell lines	63% (31/49)
10	23.026	36	7	26s Proteasome,BCL2,CREB1,F2RL1,IFNA2,IL1RN,TGFA	22	7	binding of leukocytes	24% (12/49)
11	22.687	55	11	26s Proteasome,Calcineurin protein(s),CD38,EIF4E,F2RL1,	37	7	migration of macrophages	23% (18/77)
12	21.651	23	5	CIITA,EBI3,IL27,PARP9,PDCD1	12	6	activation of lymphatic system cells	53% (16/30)
13	21.355	41	6	F2RL1,IL1RN,miR-155-5p (miRNAs w/seed UAAUGCU),	28	7	cell viability of mononuclear leukocytes	36% (15/42)
14	20.788	42	5	F2RL1,IL1RN,Pkc(s),TNFSF11,VEGFA	28	9	adhesion of immune cells	47% (21/45)
15	20.715	50	7	BTNL2,CIITA,Ifn,Ifnar,IL27,SYVN1,TGM2	33	10	activation of leukocytes	20% (14/70)
16	19.856	54	8	Ap1,CAMP,CSF2,EIF2AK2,F2RL1,IL1RN,miR-155-5p	39	7	chemotaxis of granulocytes	38% (21/56)
17	19.73	30	3	CAMP,miR-155-5p (miRNAs w/seed UAAUGCU),PSMD10	19	8	cell death of connective tissue cells	33% (8/24)
18	19.1	50	8	F2,F2RL1,IL17A,MIF,mir-1,PPRC1,REL,TGFA	35	7	cell viability of lymphatic system cells	46% (26/56)
19	18.764	67	13	Ap1,BCR (complex),CAMP,CSF2,IL12 (complex),IL21,STAT1,	48	6	synthesis of reactive oxygen species	41% (32/78)
20	18.475	41	7	F2RL1,IL17A,LDL,mir-1,PPRC1,REL,RELA	27	7	cell viability of mononuclear leukocytes	39% (19/49)
21	18.429	75	8	CCL5,F2RL1,IL1RN,miR-155-5pPSMD10,STAT4,TGFA	49	18	apoptosis of fibroblast cell lines	31% (45/144)
22	17.098	34	6	F2RL1,Igm,IL1RN,IL6,STAT3,VEGFA	23	5	binding of myeloid cells	37% (11/30)
23	16.585	33	7	CEBPA,EGF,FLT3LG,IL17A,MIF,mir-1,REL	21	5	NK cell proliferation	37% (13/35)
24	16.44	50	7	CAMP,F2RL1,IL17A,JUN,LDL,NRG1,TGFA	37	6	activation of antigen presenting cells,	50% (21/42)
25	15.167	50	7	CAMP,ETS1,F2,F2RL1,IL17A,MIF,TGFA	36	7	accumulation of cells	55% (27/49)
26	14.732	52	8	26s Proteasome,CSF1,IFNG,IL17A,IRF4,LDL,RELA,TGFA	39	5	chemotaxis of kidney cell lines	43% (17/40)
27	14.467	47	5	26s Proteasome,AKT1,LDL,TGFA,TGM2	37	5	cellular homeostasis	48% (12/25)
28	12.928	70	11	26s Proteasome,APP,CREB1,CSF1,IFNA2,IFNG,IL17A,TGFA	54	5	translation of mRNA	44% (24/55)
29	12.667	50	5	CEBPA,F2RL1,IL1RN,TNFSF11,VEGFA	36	9	quantity of IgG,recruitment of cells	31% (14/45)
30	12.33	50	7	CAMP,EIF2AK2,F2RL1,HRAS,IL17A,IL1RN,STAT2	37	6	homing of neutrophils,recruitment of cells	40% (17/42)
31	12.221	76	6	CD40LG,GAST,miR-155-5p,TNFSF11	63	7	production of reactive oxygen species	45% (19/42)
32	11.939	32	6	CAMP,ETS1,IL17A,KITLG,miR-155-5,miR-21-5p	22	4	infiltration by myeloid cells	38% (9/24)
33	11.839	34	4	BTNL2,Hbb-b2,Ifnar,TRIM24	24	6	diabetes mellitus,hypersensitive reaction	8% (2/24)
34	10.818	46	5	CEBPA,EGF,FLT3LG,IL17A,MIF	35	6	cell viability of tumor cell lines	43% (13/30)
35	9.707	21	5	F2,F2RL1,IL1RN,IL6,VEGFA	13	3	migration of antigen presenting cells	60% (9/15)
36	8.693	13	4	CD3,F2RL1,IL1RN,VEGFA	7	2	binding of myeloid cells	25% (2/8)
37	8.521	22	5	26s Proteasome,FOXO3,IL18,Pkc(s),TNFSF11	15	2	response of lymphatic system cells	60% (6/10)
38	8.01	74	8	A2M,CD40LG,GAST,mir-17,miR-17-5p,other miRNAs	58	8	anemia,binding of tumor cell lines	28% (18/64)
39	7.649	36	5	GAST,PARP9,PIK3R1,SOX4,TGFA	26	5	anemia,autophagy,organismal death	16% (4/25)
40	7.464	87	13	CD40LG,EP300,ERG,Igm,IL7,miR-19b-3p,miR-291a-3	69	5	cell death of fibroblast cell lines	28% (18/65)
41	7.181	14	6	CSF2,EDN1,F2,IL1B,KITLG,SPI1	7	1	migration of granulocytes	33% (2/6)
42	6.791	26	5	EDN1,F2,PRKCA,TNFSF11,VEGFA	17	4	Nephritis,synthesis of eicosanoid	40% (8/20)
43	6.633	17	3	IRF5,miR-155-5p (miRNAs w/seed UAAUGCU),PSMD10	11	3	apoptosis of connective tissue cells	0% (0/9)
44	6.379	18	3	ETS1,GFI1,PRL	13	2	quantity of hematopoietic progenitor cells	100% (6/6)
45	6.306	22	3	miR-155-5p (miRNAs w/seed UAAUGCU),miR-21-5p	17	2	cell death of connective tissue cells	17% (1/6)
46	6.183	27	3	CREB1,IFNA2,PDCD1	22	2	activation of leukocytes	67% (4/6)
47	5.667	14	1	GFI1	9	4	HIV infection,proliferation of blood cells	75% (3/4)
48	5.345	19	1	IL5	14	4	inflammation of body cavity	50% (2/4)
49	5.292	34	4	CAMP,CSF2,IFNG,IL12 (complex)	28	2	synthesis of leukotriene	75% (6/8)
50	4.907	17	3	EGF,PRDM1,SMARCA4	12	2	endocytosis,phagocytosis of cells	17% (1/6)
51	4.276	18	2	GFI1,Pkc(s)	14	2	differentiation of mononuclear leukocytes	50% (2/4)
52	4.199	37	3	IL2,IL21,IL4	30	4	apoptosis of connective tissue cells	42% (5/12)
53	4.16	17	3	CAMP,CSF1,Immunoglobulin	13	1	mobilization of Ca2+	67% (2/3)
54	3.889	12	2	mir-8,miR-92a-3p (and other miRNAs w/seed AUUGCAC)	8	2	cell cycle progression	0% (0/4)
55	3.13	8	1	FOXO1	5	2	hyperplasia of lymphoid organ,	0% (0/2)
56	3.024	11	3	Igm,Interferon alpha,STAT1	7	1	apoptosis of kidney cell lines	0% (0/3)
57	3	13	3	CEBPA,IFN Beta,mir-223	9	1	production of protein	33% (1/3)
58	2.236	8	1	mir-223	5	2	Bacterial Infections,production of protein	0% (0/2)
59	1.789	7	1	E2F1	5	1	cell death of fibroblasts	100% (1/1)
60	1.789	7	1	IL15	5	1	cytotoxicity of natural killer cells	100% (1/1)
61	1.789	7	1	IL1B	5	1	binding of lymphatic system cells	100% (1/1)
62	1.732	5	1	CD28	3	1	hyperplasia of lymphoid organ	0% (0/1)
63	1.508	13	1	TP53	11	1	catabolism of protein	100% (1/1)
64	0.802	17	2	HRAS,TCR	14	1	expression of mRNA	0% (0/2)
65	0.577	32	4	IFNA2,IRF7,TGFB1,TNF	27	1	systemic lupus erythematosus	25% (1/4)
66	-2.714	13	1	IL4	11	1	infection of cells	100% (1/1)
67	-4.082	8	1	miR-21-5p (and other miRNAs w/seed AGCUUAU)	6	1	cell death	100% (1/1)
68	-6.5	6	1	TCF7L2	4	1	apoptosis of fibroblast cell lines	0% (0/1)
69	-16.743	5	1	TRAP1	3	1	synthesis of reactive oxygen species	100% (1/1)
70	-23.519	58	1	APP	56	1	cancer	100% (1/1)

### “Upstream analysis” affected by δ-tocotrienol feeding to hepatitis C patients

The most interesting results of present IPA was “upstream analysis” of δ-tocotrienol treated hepatitis C patients. There were 934 gene regulators identified in this section. The 57 genes regulator correspond to present study were up-regulated (Table 7), and 64 gene regulators down-regulated (Table 8). There were several miRNAs (38), which were up-regulated and remaining other important biomarkers gene were down-regulated (Table 8). The activation Z-Scores (3.79–1.26) and *P*-values (5.39E-8 – 1.26) were significant from each biomarkers. The down-regulated biomarkers included several cytokines (IL-2, Il-5, IL-6, IL-7, IL-12, IL-13, IL-15, IL-17, IL-17A, IL-18, IL-21, IL-24, IL-27, IL-32), as well as miRNA-15, miRNA-124, miRNA-218-5P, interferon β-1a, interferon γ, TNF-α, STAT2, NOX1, prostaglandin J2, NF-κB, IκB, and TCF3 (transcription regulator), with significant activation Z-Score (− 4.56–2.531), and *P*-values were 9.17–14.00; *P* < 0.05, respectively (Table 8).

**Table 7:** Effect of δ-tocotrienol on up-regulation of fold change expression in “upstream regulator” section (57) of IPA analysis in hepatitis C patients

Upstream Regulator		Molecule Type	Predicted Activation State	Activation Z-Score	*P*-value of overlap	Mechanistic Network
#	Up-regulated					
1	miR-17-5p (and other miRNAs w/seed AAAGUGC)	mature microrna	Activated	3.798	5.39E-08	127 (7)
2	miR-155-5p (miRNAs w/seed UAAUGCU)	mature microrna	Activated	4.518	9.04E-06	137 (7)
3	miR-19b-3p (and other miRNAs w/seed GUGCAAA)	mature microrna	Activated	2.198	0.00017	
4	miR-92a-3p (and other miRNAs w/seed AUUGCAC)	mature microrna	Activated	2.187	0.00744	
5	miR-214-3p (and other miRNAs w/seed CAGCAGG)	mature microrna			0.0113	
6	miR-291a-3p (and other miRNAs w/seed AAGUGCU)	mature microrna	Activated	2.994	0.017	
7	miR-21-5p (and other miRNAs w/seed AGCUUAU)	mature microrna	Activated	2.595	0.0159	
8	miR-330-5p (and other miRNAs w/seed CUCUGGG)	mature microrna			0.0113	
9	miR-122-5p (miRNAs w/seed GGAGUGU)	mature microrna	Activated	2.586	0.0279	
10	miR-2682-5p (and other miRNAs w/seed AGGCAGU)	mature microrna			0.00584	
11	miR-205-5p (and other miRNAs w/seed CCUUCAU)	mature microrna			0.0325	
12	miR-200b-3p (and other miRNAs w/seed AAUACUG)	mature microrna		1.960	0.0273	
13	miR-542-3p (miRNAs w/seed GUGACAG)	mature microrna			0.0363	
14	miR-221-3p (and other miRNAs w/seed GCUACAU)	mature microrna		1.957	0.0349	
15	miR-147a (miRNAs w/seed UGUGUGG)	mature microrna			0.0448	
16	miR-450a-5p (and other miRNAs w/seed UUUGCGA)	mature microrna			0.0448	
17	miR-216a-5p (miRNAs w/seed AAUCUCA)	mature microrna			0.0448	
18	miR-504-5p (and other miRNAs w/seed GACCCUG)	mature microrna			0.0448	
19	miR-657 (miRNAs w/seed GCAGGUU)	mature microrna			0.0448	
20	mir-17	microrna	Activated	2.581	0.00091	
21	mir-122	microrna	Activated	3.300	0.00189	
22	mir-19	microrna	Activated	2.204	0.002	
23	mir-1	microrna	Activated	2.72	0.00354	128 (6)
24	mir-214	microrna			0.00906	
25	mir-326	microrna			0.0113	
26	mir-138	microrna			0.0113	
27	mir-32	microrna			0.0113	
28	mir-155	microrna		1.965	0.00691	173 (8)
29	mir-148	microrna		1.997	0.00103	
30	mir-199	microrna			0.0028	164 (7)
31	mir-218	microrna			0.0183	
32	mir-515	microrna			0.0225	
33	mir-132	microrna			0.0349	
34	mir-10	microrna	Activated	2.786	0.0366	
35	mir-8	microrna	Activated	2.128	0.0344	
36	mir-25	microrna		1.972	0.0349	
37	mir-622	microrna			0.0448	
38	mir-181	microrna		0.988	0.0498	
39	Immunoglobulin	complex	Activated	2.345	0.00024	283 (16)
40	prednisolone	chemical drug		1.763	0.00025	235 (13)
41	26s Proteasome	complex	Activated	2.921	0.000933	326 (16)
42	IgG	complex		1.003	0.00824	295 (16)
43	TRAP1	enzyme	Activated	2.236	0.0169	
44	IL1RN	cytokine	Activated	3.235	0.0275	
45	prostaglandin A1	chemical - endogenous non-mammalian		0.686	0.00249	159 (8)
46	AGTR1	g-protein coupled receptor		1.067	0.0291	
47	MAPK1	kinase		1.017	0.0361	
48	Ubiquitin	group			0.039	
49	IL18RAP	transmembrane receptor			0.0363	
50	TAB1	enzyme		1.258	0.0349	
51	eIF2B	complex			0.0448	
52	SNRPN	other			0.0448	
53	SNORD21	other			0.0448	
54	SOS2	other			0.0448	
55	IL1RL2	transmembrane receptor			0.0469	
56	IL18BP	other			0.0469	
57	IL10RA	transmembrane receptor	Activated	2.688	0.229

**Table 8:** Effect of δ-tocotrienol on down-regulation of fold change expression in “upstream regulators” section (64) of IPA analysis in hepatitis C patients

#	Upstream Regulator	Molecule Type	Predicted Activation State	Activation z-score	*p*-value of overlap	Mechanistic Network
Down-regulated						
1	interferon beta-1a	biologic drug			9.17E-14	
2	IL2	cytokine	Inhibited	-4.562	2.23E-09	297 (17)
3	IL15	cytokine	Inhibited	-2.247	1.37E-08	299 (19)
4	FAS	transmembrane receptor		-1.461	3.94E-08	263 (17)
5	TNF	cytokine	Inhibited	-5.914	0.000000294	378 (19)
6	IL21	cytokine	Inhibited	-2.747	0.00000339	264 (15)
7	GATA1	transcription regulator		-0.822	0.00000497	243 (11)
8	IRF1	transcription regulator	Inhibited	-3.223	0.000011	245 (13)
9	EGF	growth factor	Inhibited	-5.15	0.0000204	303 (15)
10	TGFB1	growth factor	Inhibited	-3.491	0.00004	350 (17)
11	IL6	cytokine	Inhibited	-3.043	0.0000566	284 (15)
12	IL5	cytokine	Inhibited	-4.866	0.0000654	243 (13)
13	Interferon alpha	group	Inhibited	-4.069	0.000154	150 (9)
14	STAT4	transcription regulator	Inhibited	-4.536	0.000489	111 (6)
15	IL7	cytokine	Inhibited	-2.665	0.00064	243 (18)
16	IL13	cytokine		-1.516	0.000806	295 (16)
17	STAT1	transcription regulator	Inhibited	-4.582	0.000877	241 (14)
18	IL1B	cytokine	Inhibited	-4.367	0.000982	330 (17)
19	STAT2	transcription regulator	Inhibited	-2.219	0.00105	173 (9)
20	PARP9	enzyme	Inhibited	-2.200	0.00123	142 (6)
21	FOXC1	transcription regulator		-1.961	0.002	
22	IL2RG	transmembrane receptor		-0.113	0.00233	
23	IL12 (complex)	complex	Inhibited	-2.378	0.00251	246 (17)
24	TGFA	growth factor	Inhibited	-2.888	0.00327	283 (17)
25	CD14	transmembrane receptor		-1.768	0.00332	298 (16)
26	TNFSF10	cytokine		-1.376	0.00477	297 (17)
27	mir-223	microrna	Inhibited	-2.060	0.00527	167 (7)
28	IL27	cytokine	Inhibited	-2.937	0.00527	317 (16)
29	beta-estradiol	chemical - endogenous mammalian	Inhibited	-4.574	0.00546	358 (17)
30	IL10	cytokine		-0.803	0.00582	247 (17)
31	ADORA2A	g-protein coupled receptor	Inhibited	-2.365	0.00599	175 (9)
32	IFNL1	cytokine	Inhibited	-2.925	0.00622	224 (11)
33	IL18	cytokine	Inhibited	-2.26	0.00701	326 (19)
34	NOX1	ion channel		-1.951	0.00741	263 (14)
35	SOX4	transcription regulator	Inhibited	-3.033	0.00834	
36	prostaglandin J2	chemical - endogenous non-mammalian		-1.432	0.0115	
37	E2F1	transcription regulator	Inhibited	-2.081	0.0142	
38	CREB1	transcription regulator	Inhibited	-3.766	0.0143	
39	IGF1	growth factor	Inhibited	-2.385	0.0158	
40	IL12 (family)	group		-0.500	0.016	
41	IRF5	transcription regulator	Inhibited	-2.155	0.0162	
42	FOXO4	transcription regulator		-1.98	0.0179	
43	PGF	growth factor		-1.959	0.0237	
44	BTG2	transcription regulator		1.165	0.0239	
45	mir-15	microrna		-0.927	0.0279	
46	STAT5A	transcription regulator		-0.896	0.0294	
47	NFE2L2	transcription regulator	Inhibited	-3.644	0.0295	
48	MIF	cytokine	Inhibited	-2.642	0.0304	
49	FGF10	growth factor	Inhibited	-2.200	0.0305	
50	miR-26a-5p (and other miRNAs w/seed UCAAGUA)	mature microrna		1.916	0.0309	
51	NOX4	enzyme		-1.941	0.0309	
52	NFKBIB	transcription regulator		-1.400	0.0331	
53	IFNA1/IFNA13	cytokine		-1.77	0.0331	
54	FLT3LG	cytokine	Inhibited	-2.411	0.0331	
55	IL17F	cytokine		-1.917	0.0349	
56	IL32	cytokine		-1.15	0.0416	
57	CCL5	cytokine	Inhibited	-2.621	0.042	
58	IL17A	cytokine	Inhibited	-3.075	0.0422	
59	MIR124	group		1.941	0.0435	
60	miR-218-5p (and other miRNAs w/seed UGUGCUU)	mature microrna			0.0443	
61	CXCR4	g-protein coupled receptor		-0.842	0.0447	
62	CD38	enzyme	Inhibited	-3.429	0.0482	
63	IL24	cytokine		-0.277	0.0498	
64	TCF3	transcription regulator	Inhibited	-2.530	0.231	

### “Diseases or functions annotation” affected by δ-tocotrienol feeding in hepatitis C patients

The effect of δ-tocotrienol on gene expression in “diseases or functions annotation” of IPA of mRNAs sample of chronic hepatitis C patients resulted in determining 500 types of diseases and functions. Out of these 11 type genes of diseases and functions were up-regulated, while 49 were down regulated (Table 9A and B). The up-regulated genes (11) of functions include cell death/survival cell death, organismal injury and abnormalities, cellular function and maintenance, gene expression, protein synthesis, metabolic disease, and neurological diseases as shown in Table 9A. Their *p*-values and activation Z-Scores varied from 3.94E21–8.54E6 2.64–0.71 (***P*** < 0.01), respectively (Table 9A). The gene expression of 49 were down-regulated after δ-tocotrienol treatment of chronic hepatitis C patients. These genes are involved in cellular development, cellular growth, proliferation hematology, infectious diseases, cell-to-cell signaling/interaction, cardiovascular disease, antimicrobial response, cell morphology, inflammatory response, neurological disease, humoral immune response, free radical scavenging, immunological diseases, lipid metabolism, gene expression, cancer, RNA post-transcriptional modification and many other diseases as outlined in Table 9B.

**Tables 9:** Effect of δ-tocotrienol on "diseases or functions annotation" section of IPA analysis of total mRNAs of hepatitis C patients

#	Categories	Diseases or Functions Annotation	*P*-Value	Predicted Activation	Act Z-Score	Molecules	# Molecules
A	Up-regulated (11)						
1	Cell Death and Survival	cell death	3.94E-21	Increased	2.645	ABCD1,ABL1,ACO2	349
2	Cancer, Cell Death and Survival	necrosis of malignant tumor	4.75E-21	Increased	3.412	ABL1,B2M,BCL2L11	76
3	Cellular Function and Maintenance	function of lymphatic system cells	2.1E-16		0.273	ABL1,ARHGEF,	60
4	Cellular Function and Maintenance	function of leukocytes	1.25E-15		0.051	ARHGEF6,ARRB2,B2M	77
5	Gene Expression, Protein Synthesis	translation of mRNA	1.6E-12	Increased	2.941	BTG2,DNAJC1,EIF2S3	36
6	Gene Expression	expression of mRNA	3.44E-12	Increased	2.115	BTG2,CD47,DNAJC1	43
7	Metabolic Disease	glucose metabolism disorder	2.76E-08		1.558	ABHD16A,ALOX5AP,ANAPC13	136
8	Organismal Survival	organismal death	0.00000495	Increased	11.544	ABL1,ADORA2A,APRT	210
9	Cancer, Hematological Disease	lymphoproliferative malignancy	0.00000592		1.725	ABL1,ADORA2A,AIMP1	203
10	Neurological Disease, Organismal	disorder of basal ganglia	0.0000781		1.538	ABCD1,ABL1,ADORA2A	76
11	Cancer, Organismal Injury	carcinoma	0.0000854		0.711	ABCD1,ABHD16A,ABL1	749
B	Down-regulated (49)						
12	Cellular Development, Cellular	proliferation of immune cells	1.29E-24	Decreased	-2.128	ABL1,ADORA2A,ARHGEF6	128
13	Cellular Development, Cellular	proliferation of mononuclear leukocytes	6.29E-24	Decreased	-2.073	ABL1,ADORA2A,ARHGEF6	123
14	Infectious Diseases	Viral Infection	6.4E-24	Decreased	-5.928	ABL1,ADORA2A,AGO4	207
15	Cellular Growth and Proliferation	proliferation of lymphatic system cells	8.63E-24	Decreased	-2.019	ABL1,ADORA2A,ARHGEF6	129
16	Immunological Disease	systemic autoimmune syndrome	2.37E-23		-0.774	ABHD16A,ADORA2A,AKR1D1	163
17	Hematological System Development	quantity of mononuclear leukocytes	6.64E-19	Decreased	-4.691	ABL1,ADORA2A,ARHGEF6	113
18	Lymphoid Tissue Structure	quantity of lymphatic system cells	1.46E-18	Decreased	-4.679	ABL1,ADORA2A,ARHGEF6	115
19	Hematological System Development	quantity of blood cells	6.22E-16	Decreased	-4.724	ABL1,ADD3,ADORA2A	134
20	Cell-To-Cell Signaling and Interaction	activation of cells	2E-15	Decreased	-5.698	ADORA2A,AFP,ARRB2	127
21	Connective Tissue Disorders	inflammation of joint	2.16E-13		-1.573	ABL1,ADORA2A,AKR1D1	128
22	Cardiovascular Disease, Developmental	Diamond-Blackfan anemia	4.55E-11			CD52,FLVCR1,RPL11	13
23	Antimicrobial Response, Inflammatory	antimicrobial response	8.55E-09		-1.395	APOBEC3A,ATG5,BCL10	44
24	Embryonic Development, Hematological	formation of lymphoid tissue	1.45E-08	Decreased	-2.618	ABL1,B2M,BCL2L11	48
25	Free Radical Scavenging	metabolism of reactive oxygen species	1.56E-08	Decreased	-2.89	ABL1,ATG5,ATP7A	63
26	Neurological Disease, Skeletal	neuromuscular disease	5.12E-07		-0.200	ABL1,ADORA2A,ALAS1	95
27	Cell Morphology	morphology of blood cells	7.37E-07			ABCD1,ABL1,ADD3	52
28	Inflammatory Response, Neurological	inflammation of central nervous system	0.00000109		-1.099	ADORA2A,B2M,C3AR1	48
29	Humoral Immune Response, Protein	production of antibody	0.00000114		-1.497	B2M,BCL10,BCL2L11	40
30	Endocrine System Disorders	diabetes mellitus	0.00000166	Decreased	-2.058	ABHD16A,ALOX5AP,ANAPC13	110
31	Digestive System Development	morphology of Peyer's patches	0.00000208			DDX58,ID2,IGKC	12
32	Cellular Compromise, Inflammatory	degranulation of cells	0.0000021	Decreased	-3.08	C3AR1,C5AR1,CAMP	31
33	Cell Signaling, Molecular Transport	mobilization of Ca2+	0.00000212	Decreased	-2.95	ADORA2A,ARRB2,B2M	42
34	Cell-To-Cell Signaling and Interaction	binding of leukocytes	0.00000273	Decreased	-4.799	ABL1,ADORA2A,ARRB2	46
35	Immunological Disease	allergy	0.00000286		-1.655	ABL1,ACO2,ADORA2A	49
36	Humoral Immune Response, Protein	quantity of immunoglobulin	0.00000494		-1.731	B2M,BCL10,BCL2L11	37
37	RNA Post-Transcriptional Modification	processing of RNA	0.0000059		-0.670	ADAT1,AFF2,CELF1	36
38	Hematological System Development	quantity of thymocytes	0.00000592	Decreased	-3.599	ABL1,B2M,BCL10	30
39	Immunological Disease	abnormal morphology of immune	0.00000593			ABCD1,ABL1,B2M	37
40	Cancer, Hematological Disease	mature B-cell lymphoma	0.00000888			ABL1,B2M,BCL10	38
41	Digestive System Development	abnormal morphology of Peyer's	0.00000906			DDX58,ID2,IGKC	11
42	Lipid Metabolism, Small Molecule	synthesis of eicosanoid	0.00000989	Decreased	-3.209	ALOX5AP,ATP5J,C5AR1	29
43	Cellular Growth and Proliferation	expansion of cells	0.0000113		-0.717	ADORA2A,B2M,BMI1	37
44	Lipid Metabolism, Small Molecule	synthesis of leukotriene C4	0.0000148	Decreased	-2.753	ALOX5AP,C5AR1,COTL1	8
45	Gene Expression	activation of DNA endogenous	0.000016	Decreased	-3.846	ARRB2,ATF4,BMI1	111
46	Antigen Presentation, Inflammatory	antigen presentation	0.0000715		-1.556	ARL8B,CD74,CST3	14
47	Cell Death and Survival, Organismal	cell death of kidney cells	0.0000715		-1.863	ATG5,ATP1A1,BCL10	39
48	Cellular Movement, Hematological	chemotaxis of granulocytes	0.0000723	Decreased	-2.235	ADORA2A,BST1,C3AR1	24
49	Cancer, Hematological Disease	large-cell lymphoma	0.0000741			B2M,BCL2L11,CAMLG	34
50	Cell-To-Cell Signaling and Interaction	binding of mononuclear leukocytes	0.0000753	Decreased	-3.212	CD47,CD48,CD58	25
51	Cellular Movement, Embryonic	chemotaxis of embryonic cell lines	0.0000767	Decreased	-2.587	ARRB2,CAMP,CXCL1	7
52	Cellular Movement, Hair and Skin	chemotaxis of epithelial cell lines	0.0000767	Decreased	-2.587	ARRB2,CAMP,CXCL1	7
53	Cell Death and Survival, Skeletal	cell death of smooth muscle cells	0.0000775		-0.332	ARRB2,CAMP,CASP3	16
54	Cell Death and Survival	cell viability of phagocytes	0.0000775	Decreased	-2.939	BCL2A1,CD48,CEBPB	16
55	Cell Death and Survival	killing of lymphatic system cells	0.0000789	Decreased	-2.016	BCL2L11,CD47,CD48	10
56	Cell Death and Survival	cell viability of mononuclear leukocytes	0.0000805	Decreased	-3.491	ATG3,BCL10,BCL2L11	25
57	Cellular Development, Cellular Growth	differentiation of myeloid leukocytes	0.0000809		-1.081	ABL1,CAMP,CD47	31
58	Cell-To-Cell Signaling and Interaction	binding of lymphatic system cells	0.0000847	Decreased	-3.360	CD47,CD48,CD58	23
59	RNA Post-Transcriptional Modification	unwinding of mRNA	0.000086			EIF4A1,EIF4A2,EIF4B	3
60	Cell Death and Survival, Organismal	cell death of epithelial cells	0.000136		-1.105	ARRB2,ATG5,BCL10	51

The results described so far are summarized in Table 10. The data were divided into 12 categories, each category has 5 topics (total 60), and out of these 60 topics, only 13 topics were further investigated in detail for their functions related to present studies. For example, the “diseases and disorder” category (III) includes infectious diseases, immunological diseases, cancer, and organismal injury/abnormalities and tumor morphology (Table 10). The “molecular and cellular functions” category (IV) includes cellular development, cellular growth and proliferation, death/survival, cell-to-cell signal ligand interaction and cellular function and maintenance. Table 10 also includes a list of expression log ratio of 10 up-regulated genes (SNORD15A, SNORA32, SNORA56, SNORA9, SNORA3B, SNORA3A, HIST1H2AD, LINC00305, HHIPL2), and 10 down-regulated genes (HMGN1P3, SNHG25, SNORA67, RPL17-C18orf32, ISY1-RAB43, ARHGEF18, KLRC4-KLRK1/KLRK1, HIST1H3J, MTHFS, SNORA16A) were related to present investigation. At the end, out of 360 “canonical pathways” of IPA of total mRNAs samples of effects of δ-tocotrienol treatment to hepatitis C patients, 33 pathways are selected, which are associated with various signaling and biomarkers relative to present results (Table 11). The heat map (Fig. 2) also depicts same diseases and functions as outlined in Tables 9A, B and 10.

**Table 10:** Summary of IPA analyses of RNAs obtained from δ-tocotrienol treatment of hepatitis C patients

#	Subjects	*P*-Value ovrlap	Overlap	#	Subjects	*P*-Value ovrlap	# Molecules
I	Top Canonical Pathways			VII	Cardiotoxicity		
1	EIF2 Signaling	1.28E-37	30.3 % 67/221	31	Cardiac Infarction	3.62E-01 - 5.40E-04	23
2	Regulation of eIF4 and p70S6K Signaling	5.38 E-140	21.0 % 33/157	32	Caediac Necrosis/Cell Death	1.65E-01 - 2.56E-03	23
3	mTOR Signaling	1.28 E-13	18.4 % 37/102	33	Cardiac Dycfunction	4.31E-01 - 2.63E-03	11
4	B Cell Receptor Signaling	8.35 E-08	14.2 % 27/190	34	Cardiac Fibrosis	1.77E-01 - 5.68E-03	14
5	Signaling	1.72E-06	16.2 % 18/111	35	Cardiac Transformation	1.10E-02 - 1.10E-02	2
II	Top Upstream Regulators		Predicted Activation	VIII	Hepatotoxicity		
6	ST 1926	5.62E-20	Activated	36	Liver Proliferation	2.15E-01 - 5.85E-05	26
7	Sirolimus	2.32E-18	Activated	37	Liver Necrosis/Cell Death	6.13E-01 - 6.59E-05	29
8	CD 437	1.45E-17	Activated	38	Liver Damage	4.69E-01 - 1.81E-04	35
9	RICTOR	1.64E-17	Activated	39	Liver Inflamma/Hepatitistion	4.52E-01 - 5.02E-04	36
10	MYCN	3.22E-15	Inhibited	40	Liver Cirrhosis	4.19E-02 - 1.65E-03	21
III	Diseases and Disorder		# Molecules	IX	Nephrotoxicity		
11	Infectious Diseases	1.14E-04 - 1.29E-24	244	41	Renal Necrosis/Cell Death	3.32E-01 - 7.15E-05	46
12	Immunological Disease	7.41E-05 - 2.37E-23	372	42	Renal Inflammation	3.74E-01 - 1.69E-03	33
13	Cancer	1.25E-04 - 4.75E-22	839	43	Renal Nephritis	3.70E-01 - 1.69E-03	33
14	Organismal Injury and Abnormalities	1.36E-04 - 4.75E-21	865	44	Renal Damage	5.15E01 - 3.12E-03	21
15	Tumor Morphology	1.19E-04 - 4.75E-21	82	45	Glomerular Injury	1.00E-00 - 1.47E-02	22
IV	Molecular and Cellular Functions		# Molecules	X	Top Regulator Effect Networks	Disease & Functions	Consistency Score
16	Cellular Development	1.24E-04 - 1.29E-24	222	46	Ap1,CAMP,F2RL1,IL17A,IL1RN,KITLG,mir10,NRG1,SELP (+2 >)	Activationof antigen presenting cells (+11 >)	40.848
17	Cellular Growth and Proliferation	1.24E-04 - 1.29E-24	206	47	AP1,CAMP,EIF2AK2,F2RL1,IL17A,IL1RN, KITLG (+2 >)	Activationof phagocytes (+9 >)	36.338
18	Cell Death and Survival	1.36E -04 - 3.94E-21	371	48	26s Proteasome,ANGPT2,AP1,BCL2,CAMP,CEBPA,F2RL (+6 >)	Activationof antigen presenting cells (+10 >)	32.199
19	Cell-To-Cell Signalingand Interaction	1.34EE-18-04 - 7.04	183	49	26s Proteasome,CAMP,CSF1,IL17A,JUN,LDL (+5 >)F2RL (+6 >)	Activationof antigen presenting cells (+7 >)	30.414
20	Cellular Function and Maintenance	1.02E-04 - 2.10E-16	232	50	AP1,CAMP,CCL5,EIF2AK2,F2RL1,FGF10,IL17A,IL1RN (+5 >)	Accumulation of leukocytes (+19 >)	30.375
V	Physiological System Development and Function		# Molecules	XI	Top Networks (Associated Network Functions)		Score
21	Hematological System Development and Function	1.34E-04 -1.29E-24	255	51	Developmentall Disorder, Hereditary Disorder, Metabolic Diseases		46
22	Lymphoid Tissue Structure and Development	1.33E-04 -1.29E-24	194	52	Cancer, Cell Death and Survival, Organismal Injury and Abnormalities		44
23	Tissue Morphology	1.19E-04 - 2.45E-19	184	53	Post-Translational Midification, Cell Cycle, Cellular Development		44
24	Immune Cell Trafficking	1.34E-04 - 7.04E-18	160	54	Cancer, Hematological Disease, Immunological Disease		41
25	Hematopoiesis	1.02E004 - 6.87E-14	130	55	Protein Synthesis, RNA Post-Transcriptional Modification, Gene Expression		39
VI	Top Tox Functions (Clinical Chemistry and Hematology)		# Molecules	XII	Top Toxicology Lists	*p*-value	Overlap
26	Increased Levels of Albumin	2.38E-01 - 1.24E-02	4	56	Renal Necrosis/Cell Death	1.58E-05	8.60 % 46/538
27	Increased Levels of Alkaline Phosphatase	2.12E-01 - 4,42E-02	6	57	Liver Prolification	1.80E-05	11.0 % 26/236
28	Decreased Levels of Hematocrit	5.71E-02 - 5.71E-02	2	58	Liver Necrosis/ Cell Death	8.35E-05	9.6 % 29/303
29	Increased Levels of Hematocrit	6.20E-02 - 6,20E-02	8	59	Mechanism of Gene regulation by Peroxisome	2.74E-04	13.7 % 13/95
30	Increased Levels of Potassium	5.36E-01 - 8.64E-02	2	60	Increases Liver Damage	7.40E-04	11.4 % 15/132
A	Gene Expression Fold Change (Up-regulated)	Expression Value		B	Gene Expression Fold Change (Down-regulated)	Expression Value	
1	SNORD15A	581.151		1	HMGN1P3	-381.06	
2	SNORA32	390.353		2	SNHG25	-350.0555	
3	SNORA56	185.194		3	SNORA67	-148.69	
4	SNORA9	124.698		4	RPL17-C18orf32	-67.253	
5	SNORS3B	102.91		5	ISY1-RAB43	-51.147	
6	SNORA3A	93.09		6	ARHGEF18	-41.381	
7	HIST1H2AD	20.784		7	KLRC4-KLRK1/KLK1	-20.578	
8	SNORD3D	17.157		8	HIST1H3J	-19.795	
9	LINC00305	4.853		9	MTHFS	-18.71	
10	HHIPL2	4.844		10	SNORA16A	-18.285

**Table 11:** Effect of δ-tocotrienol on canonical pathways (33) of IPA ingenuity canonical pathways analysis (360) in hepatitis C patients

#	Ingenuity Canonical Pathways (Fold Change Expression)	-log (*p*-value)	Ratio	Z-Score	Molecules
1	EIF2 Signaling; Eukaryotic translation initiation factors (221)	36.900	0.303	-5.692	RPL7A,EIF3G,RPL13A,RPL32,RPS24,RPL37A,RPL23,RPL26,RPS13
2	Regulation of eIF4 and p70S6K signaling (157)	13.300	0.210	0.000	PPP2R5E, EIF3G, RPS26
3	Protein ubiquitination pathway (265)	3.130	0.091	#NUM!	UBE2J1, USP19, UBA52
4	mTOR signaling; Mammalian target of rapamycin (201)	12.900	0.184	-2.138	PPP2R5E, EIF3G, RPS26
5	Type I Diabetes Mellitus Signaling (111)	5.760	0.162	-2.496	NFKB1,MAP3K5,JAK2,HLA-DQB1,IFNGR2,TNFRSF1B,PIAS1,TRADD
6	Th1 and Th2 Activation Pathway (185)	5.640	0.130	#NUM!	NFKB1,JAK2,NOTCH1,HLA-DQB1,IFNGR2,PIK3R1,HLA-DRA
7	Interferon Signaling (36)	4.700	0.250	-2.333	IFNGR1,OAS1,IFIT1,JAK2,IFITM1,IFNGR2,IFITM2,PIAS1,PSMB8
8	Role of IL-17F (44)	3.960	0.205	-3.000	NFKB1,ATF4,CREB1,RPS6KA3,CXCL1,MAPK1,CXCL8,RPS6KA4
9	IL-8 Signaling (197)	3.320	0.102	-4.123	NFKB1,GNA13,GNB4,RACK1,VEGFA,MYL12B,PIK3R1,ARRB2,NCF2
10	NF-κB Signaling (181)	2.940	0.099	-4.243	GSK3B,SIGIRR,NFKB1,CSNK2B,TNFRSF1B,IL1R2,PIK3R1,TRADD
11	IL-17A Signaling in Fibroblasts (35)	2.400	0.171	#NUM!	GSK3B,NFKB1,CEBPD,CEBPB,MAPK1,TRAF6
12	IL-6 Signaling (128)	2.360	0.102	-3.051	NFKB1,JAK2,CSNK2B,TNFRSF1B,VEGFA,IL1R2,PIK3R1,CXCL8,FRS2
13	Induction of Apoptosis by HIV1 (61)	2.280	0.131	-2.828	CXCR4,NFKB1,MAP3K5,TNFRSF1B,CASP3,TRADD,RIPK1,SLC25A13
14	HMGB1 Signaling (133)	2.220	0.098	-3.606	OSM,NFKB1,IFNGR2,TNFRSF1B,PIK3R1,SP1,CXCL8,IFNGR1,HMGB1
15	PPAR Signaling (95)	2.040	0.105	1.897	NFKB1,TNFRSF1B,PTGS2,IL18RAP,MAPK1,IL1R2,HSP90AB1,SCAND1
16	IL-10 Signaling (69)	1.960	0.116	#NUM!	NFKB1,IL18RAP,MAPK1,IL1R2,SP1,FCGR2A,TRAF6,IL10RA
17	iNOS Signaling (45)	1.860	0.133	-2.449	IFNGR1,NFKB1,JAK2,IFNGR2,MAPK1,TRAF6
18	Insulin Receptor Signaling (141)	1.650	0.085	-1.508	GSK3B,PPP1CC,PTEN,JAK2,GYS1,PDE3B,FRS2,MAPK1,GSK3A
19	p53 Signaling (111)	1.600	0.090	0.000	GSK3B,DRAM1,PTEN,HIF1A,FRS2,ATR,ST13,PIK3R1,PIAS1,PCNA
20	Role of IL-17A in Arthritis (69)	1.490	0.101	#NUM!	NFKB1,FRS2,PTGS2,CXCL1,MAPK1,PIK3R1,CXCL8
21	Toll-like Receptor Signaling (76)	1.300	0.092	-1.000	SIGIRR,TLR8,UBA52,NFKB1,MAP3K1,MAPK1,TRAF6
22	IL-1 Signaling (92)	1.300	0.087	-2.449	GNAQ,NFKB1,GNA13,GNB4,RACK1,MAP3K1,MAPK1,TRAF6
23	Apoptosis Signaling (90)	0.987	0.078	-0.378	NFKB1,MAP3K5,BCL2L11,BCL2A1,TNFRSF1B,MAPK1,CASP3
24	PDGF Signaling (90)	0.987	0.078	-2.646	ABL1,JAK2,CSNK2B,MAP3K1,FRS2,MAPK1,PIK3R1
25	Type II Diabetes Mellitus Signaling (128)	0.944	0.070	-2.333	NFKB1,MAP3K5,TNFRSF1B,MAP3K1,FRS2,CEBPB,MAPK1,PIK3R1
26	IL-15 Signaling (76)	0.904	0.107	#NUM!	NFKB1,JAK2,TXK
27	autophagy (62)	0.859	0.081	#NUM!	CTSW,ATG3,ATG5,CTSC,LAMP2
28	IL-2 Signaling (64)	0.818	0.078	-2.000	CSNK2B,FRS2,MAPK1,PIK3R1,IL2RG
29	PPARα/RXRα Activation (180)	0.759	0.061	3.000	TGS1,GNAQ,TGFBR2,NFKB1,JAK2,IL18RAP,MAPK1,MED12,IL1R2
30	TNFR1 (32)	2.210	0.140	-2.646	NFKB1,MAP4K2,MAP3K1,PAK1,CASP3,TRADD,RIPK1
31	STAT3 Pathway (74)	0.641	0.068	-1.342	TGFBR2,JAK2,MAPK1,PTPN6,IGF2R
32	Nitric Oxide Signaling in the Cardiovascular System (113)	0.633	0.062	-2.646	ITPR2,VEGFA,PDE3B,FRS2,MAPK1,PIK3R1,HSP90AB1
33	Osteoarthritis Pathway (210)	3.370	0.100	-2.524	NFKB1,CREB1,NOTCH1,TNFRSF1B,VEGFA,KEF1,IL-1R2,mir-140

## Discussion

The fold-change gene expression data analyzed by Ingenuity Pathway Analysis describes cellular and biological mechanisms at the molecular level on the effect of δ-tocotrienol in chronic hepatitis C patients. It involves metabolic and cellular processes, mainly associated with catalytic activity of structural molecules. It also reveals an insight of correlation of signaling pathways and transcriptional factors, and subsequently describes inhibition or activation of anti- and pro-inflammatory genes. The results of these functional genomics produced a huge amount of data analyzed by biological networks using differentially gene expression after treatment with δ-tocotrienol to chronic hepatitis C patients. It predicts possible canonical pathways, upstream regulators, diseases and functional metabolic networks. The differential gene expressions of several biological functions illustrated in the heat map is shown in Fig. 2.

The present data revealed that genes responsible for replication of virus, infection by RNA viruses, infection of tumor cell lines, HIV infection and replication of influenza virus were all down-regulated, while cell death processes were all up-regulated. Moreover, as mentioned earlier, that Table 10 includes a list of expression log ratio of 10 up-regulated and 10 down-regulated genes. The forgoing information is mainly from “Ingenuity Knowledge Base” including as the information source for these facts and pathways.

The first up-regulated gene, SNORD15 is a non-coding RNA (ncRNA) gene which involves in the modification of other small nuclear RNAs (snRNAs), located in the nucleolus of the eukaryotic cell, which is a major site of snRNA biogenesis, and known as small nuclear RNA (snoRNA) [9]. It belongs to C/D box class of snoRNA, which function in directing site-specific 2-O-methylation of substrate RNAs [9]. In humans, there are two closely related copies of the U15 snoRNA (called SNORD15A and SNORD15B) [10]. Histone H2A type 1-D encoded by HIST1H2AD gene in humans. Histones are basic nuclear proteins that are responsible for the nucleosome structure of chromosomal fiber in eukaryotes. LINC00305 is associated with atherosclerotic plagues and monocytes [11]. Overexpression of LINC00305 promoted the expression of inflammation-associated genes in THP-1cells and reduced the expression of contractile markers in co-cultured human aortic smooth muscle cells. LINC00305 overexpression activated NF-κB and inhibition of NF-κB abolished LINC00305-mediated activation of cytokine expression [12]. HHIPL-2 identified as a candidate gene involved in iron-related modulation of osteoblast markers. The excess of iron limits HHIP-2 gene expression and decreases osteoblastic activity in human MG-63 cell [13].

Whereas, the “High Mobility group Nucleosome Domain 1 Pseudogene 3” (HMGN1P3) is a down-regulated pseudogene 3, and belongs to NURSA nuclear receptor signaling pathways expression of HMGN1P3 gene, and involves in all type of cancers (from breast, prostate, pancreas, colon kidney, lung, ovary, uterus) [14, 15]. The small nuclear RNA (SNORA67) is also a down-regulated non-coding RNA molecule that belongs to the H/ACA class of snoRNA, which guide the sites of modification of uridines and pseudouridines [16]. The ISY1-RAB43 is the naturally occurring read-through transcription gene, which act between the neighboring ISY1 (splicing factor homolog) and RAB43 (member RAS oncogene family) gene on chromosome 3. The read-through transcript encodes is a protein that shares sequence identity with the upstream gene product, but its C-terminus is distinct due to a frameshift relative to the downstream gene [17]. The Rho/Rac guanine nucleotide exchange factor 18 (ARHGEF18) is GTP binding proteins that regulate a number of cellular functions such as, cytoskeletal rearrangements, gene transcription, cell growth and motility [18].

The KLRC4-KLRK1 gene represents also naturally occurring down-regulated read-through transcription gene, which acts between the neighboring KLRK4 (killer cell lectin-like receptor subfamily C, member 4) family. This protein and its ligands are therapeutic targets for the treatment of immune diseases and cancers [19]. Histone H3.1 is a protein that in human encoded by the HIST1H3J gene [20, 21]. Histones are basic nuclear proteins that are responsible for the nucleosomes fiber in eukaryotes. The methenyltetrahydrofolate synthetase (MTHFS) is down- regulated encoded an enzyme that catalyzes the conversion of 5-formyltetrahydrofolate to 5, 10-methenyltetrahydrofolate, and helps regulate carbon flow through the folate-dependent one-carbon metabolic network [22, 23]. The small nucleolar RNA, H/ACA box 16A (SNORA16A) gene provides a unified query environment for genes defined by sequence [24].

The study also provides an insight of correlation of signaling pathways and transcriptional factors and subsequently describes the modulation of anti- as well as pro-inflammatory genes. It described the effects δ-tocotrienol in chronic hepatitis C patients on gene expression of liver cancer, liver hyperplasia, cell proliferation, cell growth, cell death/survival, infections, inflammatory diseases, and apoptosis. Collectively, the effects of δ-tocotrienol on “canonical pathways” observed in IPA of total mRNA sample of hepatitis C patients resulted in modulation of over 360 pathways, which are associated with multiple signaling pathways. It is conceivable that some or most of these pathways may be controlled by the proteasome, since the protein ubiquitination pathway was down-regulated by δ-tocotrienol treatment as described previously [1].

**Fig. 3:**
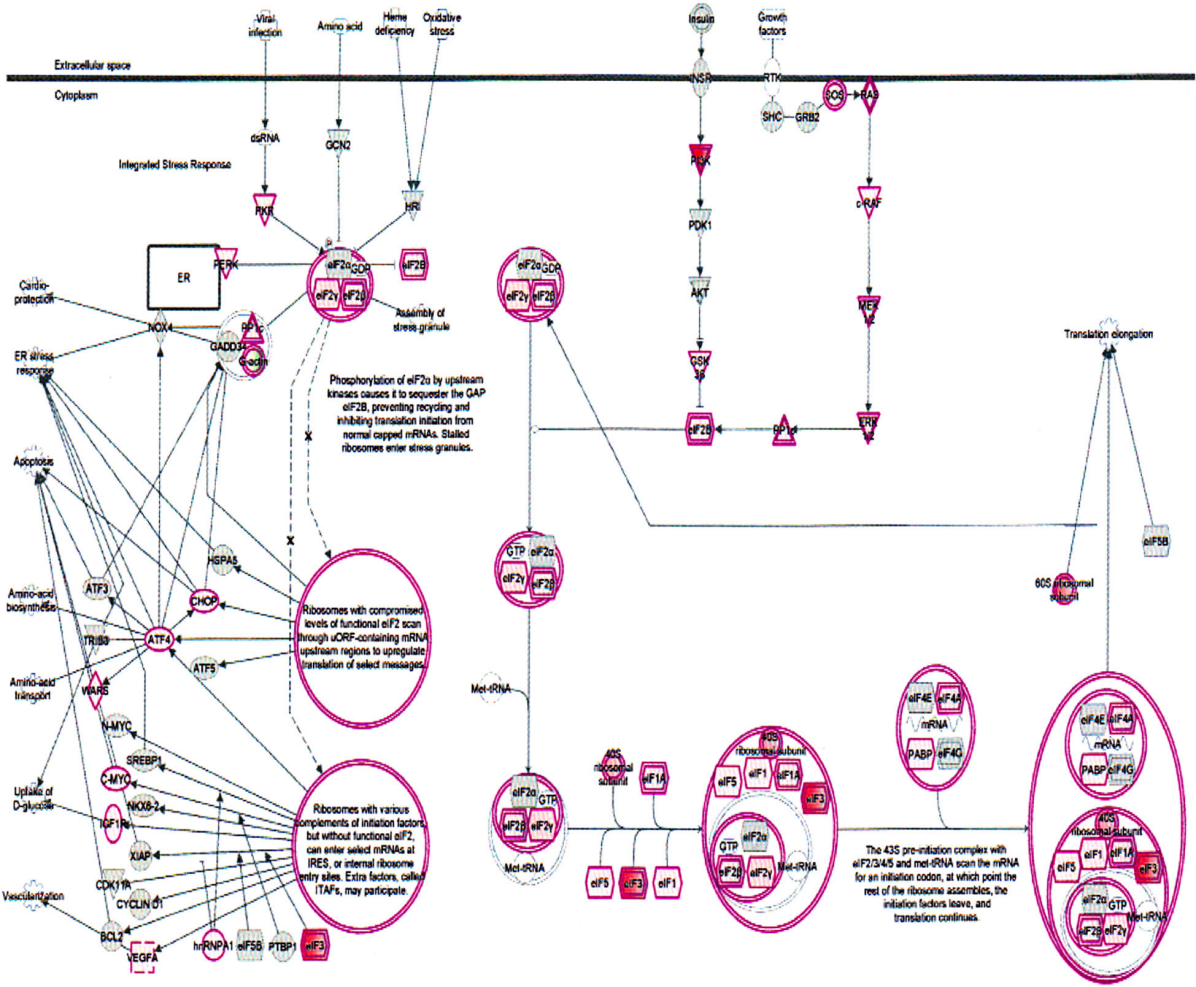
Effect on eukaryotic translation initiation factors (EIF2) signaling pathway in plasma of total mRNAs obtained from δ-tocotrienol treatment of hepatitis C patients. EIF2 was down-regulated by δ-tocotrienol treatment, which is involved in protein synthesis, requires a large number of polypeptides. EIF2 is a GTP-binding protein, which initiates specific form of met-tRNA onto the ribosome

**Fig. 4:**
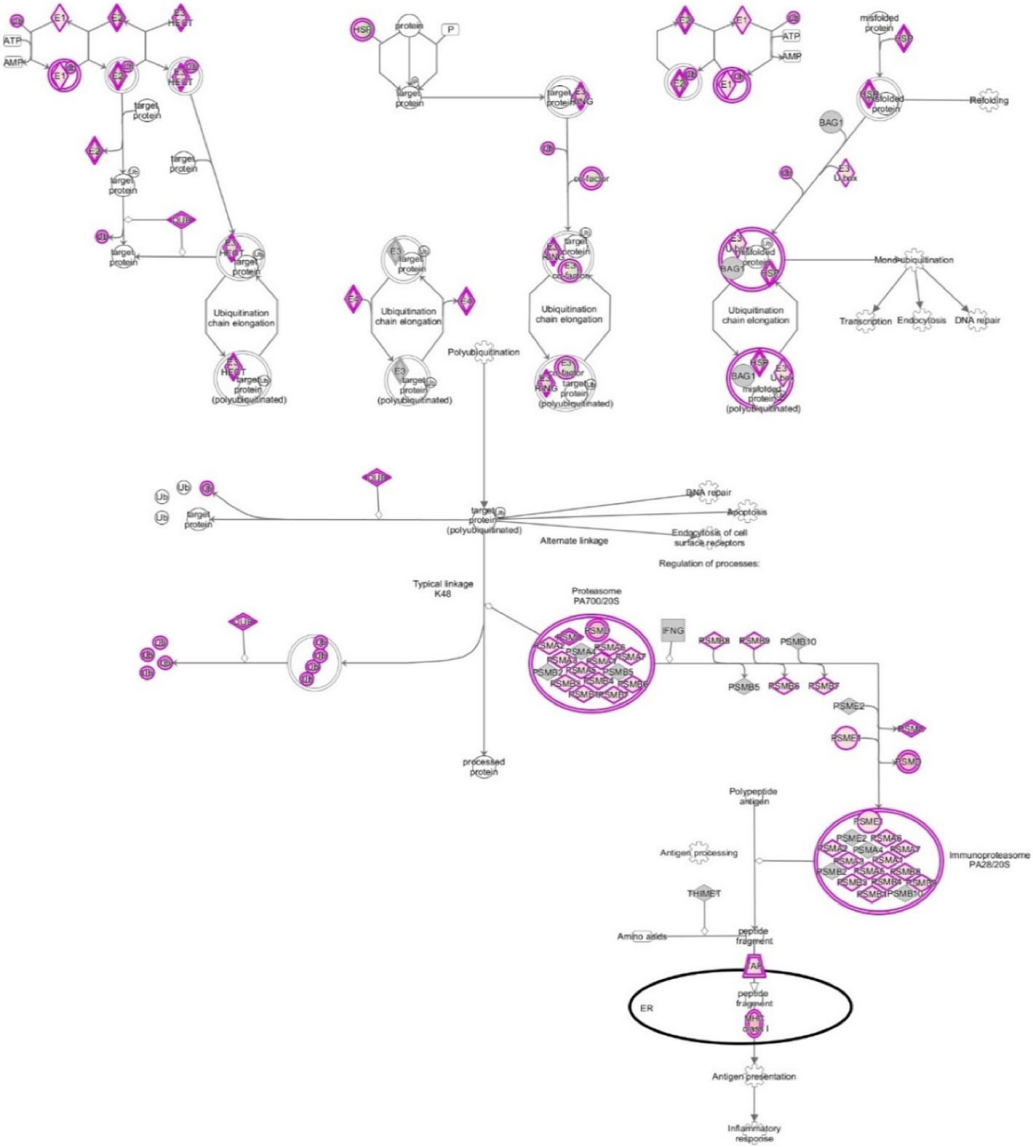
Effect on protein ubiquitination signaling pathway in plasma of total mRNAs obtained from δ-tocotrienol treatment of hepatitis C patients. The protein ubiquitination pathway was down-regulated by δ-tocotrienol treatment. It plays a major role in the degradation of regulatory proteins, including a variety of cellular processes, such as cell cycle, cell proliferation, DNA repair, apoptosis, transcription regulation, cell surface receptors, ion channel regulation and antigen presentation

The important signaling pathways modulated by tocotrienols are as follows: at the top of the list is “eukaryotic translation initiation factors” (EIF2) signaling pathway (Fig. 3). This is involved in protein synthesis, and requires a large number of polypeptides. EIF2 is a GTP-binding protein, which initiates specific forms of met-tRNA onto the ribosome. Its important function is to deliver charged initiator met-tRNA to the ribosome, it also identifies the translational starting site [9]. This is followed by protein ubiquitination pathway, which plays a major role in the degradation of short-lived or regulatory proteins. It plays a role in a variety of cellular processes, such as cell cycle, cell proliferation, apoptosis, DNA repair, transcriptional regulation, cell surface receptors, ion channels regulation and antigen presentation, as outlined in Fig. 4 [10]. We have discussed the importance of ubiquitination in our several earlier publications [11–15].

**Fig. 5:**
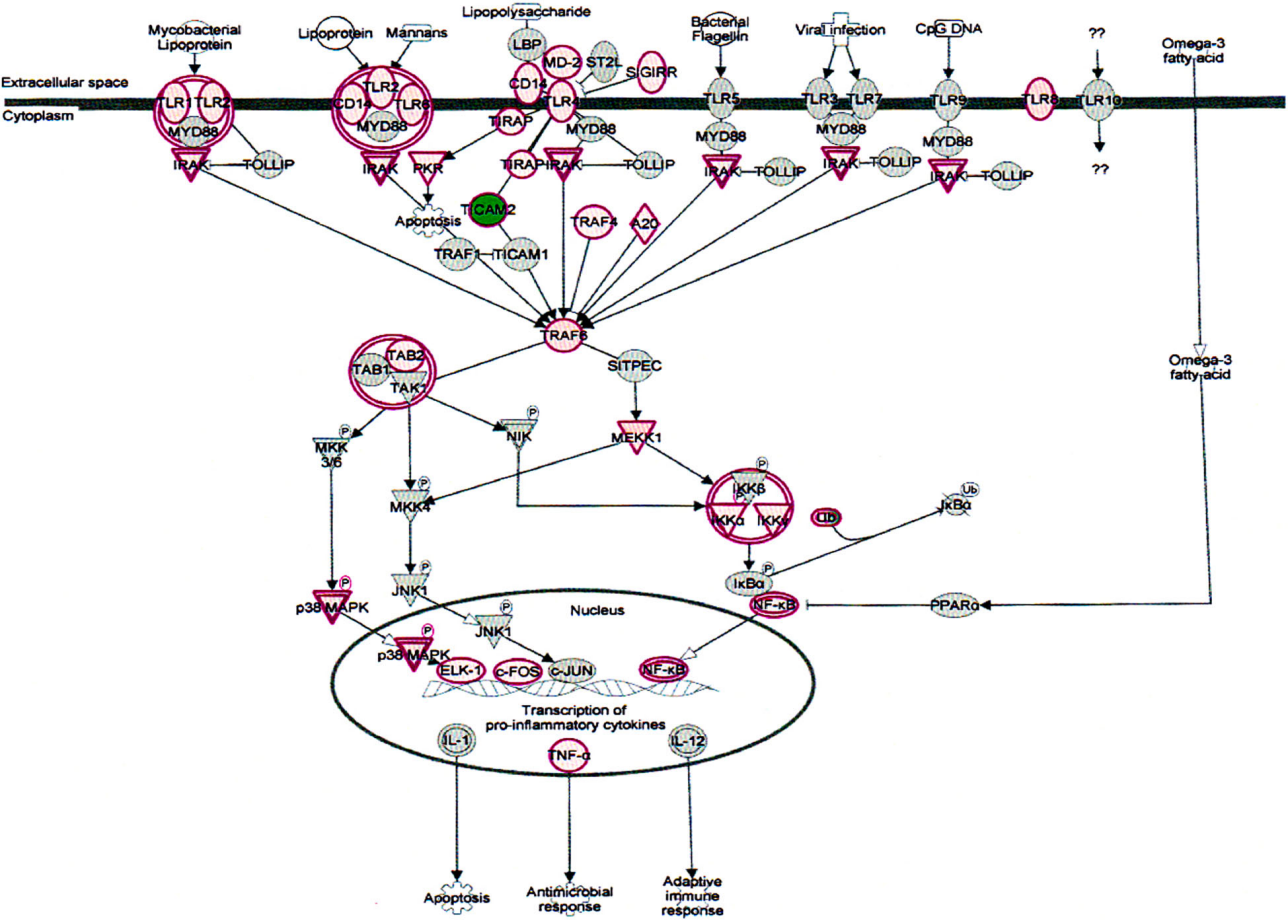
Effect on toll-like receptor (TLRs) signaling pathways in plasma of total mRNAs obtained from δ-tocotrienol treatment of hepatitis C patients. The TLRs were down-regulated by δ-tocotrienol treatment, these belong to the family of pathogen-associated receptors, and bind to a number of bacteria and viruses, such as viral DNA, lipopolysaccharide, and CpG DNA motifs. TLRs form a complex with different combinations of adapter molecules like MYD88, TRAF6 and TIRAP to initiate signal transduction upon ligand binding

δ-Tocotrienol treatment of chronic hepatitis C patients also affects several other regulators in canonical pathways, we will limit our discussion to only important signaling and biomarkers associated with present investigation. The toll-like receptor signaling (TLRs) belongs to the family of pathogen-associated pattern recognition receptors, and bind to specific molecular patterns in bacteria and viruses. The pathogen-associated ligands include bacterial flagellin, viral DNA, lipopolysaccharide (LPS) and CpG DNA motifs. TLRs form a complex with different combinations of adopter molecules like MYD88, TRAF6 and TIRAP to initiate signal transduction upon ligand binding. This binding triggers a cascade of signaling events via the TLR-adapter complex, and downstream sigling molecules like p38MAPK. JNK. NF-κB activated and translocated into the nucleus, where they activate transcription regulators like c-Fos and c-Jun, leading to the induction of several pro-inflammatory cytokines, eventually leading to antibacterial and antiviral responses [25, 26]. Tocotrienol treatment causes a downregulation of the TLR pathways in hepatitis C patients. The toll-like receptor signaling pathways outlined in Fig. 5.

**Fig. 6:**
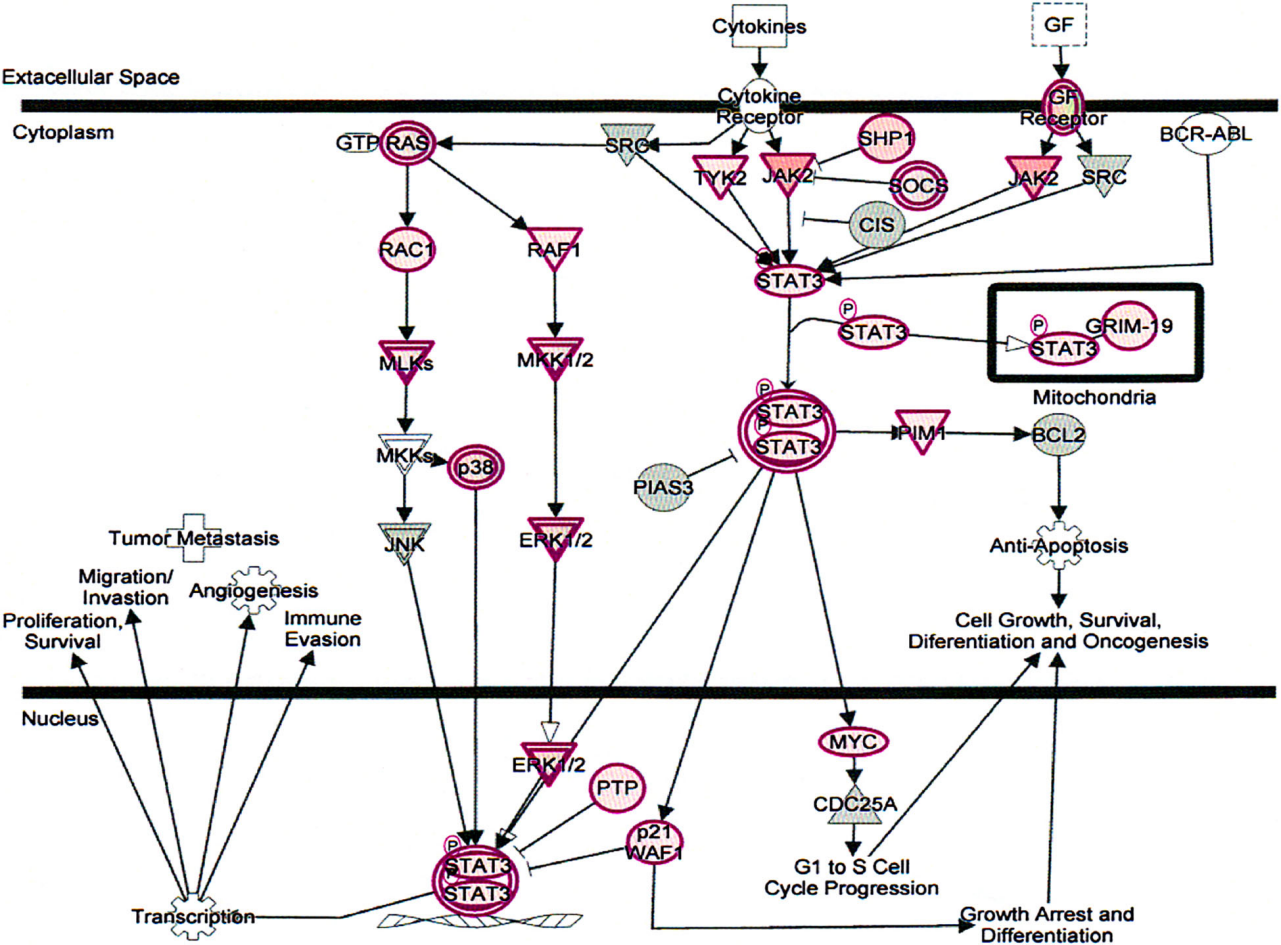
Effect on signal transducer and activators of transcription (STATs) signaling pathways in plasma of total mRNAs obtained from δ-tocotrienol treatment of hepatitis C patients. The STATs were down-regulated by δ-tocotrienol treatment, and belong to a family of cytoplasmic proteins with Src homology-2 (SH2) domains that acts as signal messenger and transcriptional factors and responses to cytokines and growth factors. The STAT pathways are activated via tyrosine phosphorylation cascade and play an important role in normal development of hematopoiesis, and regulates cancer metastasis by regulating the expression of genes that are critical to cell survival, cell proliferation, invasion, angiogenesis, and tumor immune evasion

The signal transducers and activators of transcription (STATs) are a family of cytoplasmic proteins with Src homology-2 (SH2) domains. STATs acts as a signal messenger and transcription factors. It participates in normal cellular responses to cytokines and growth factors. STATs pathways activated via tyrosine phosphorylation cascade after ligand binding by stimulation of the cytokine receptor-kinase complex and growth factor-receptor complex. The IL-6 cytokine activates STAT3 and STAT1. STAT3 encoded in human gene. The STAT3 signaling pathway (Fig. 6) plays an important role in normal development, particularly hematopoiesis, and regulates cancer metastasis by regulating the expression of genes that are critical to cell survival, cell proliferation, invasion, angiogenesis, and tumor immune evasion [27–29].

**Fig. 7:**
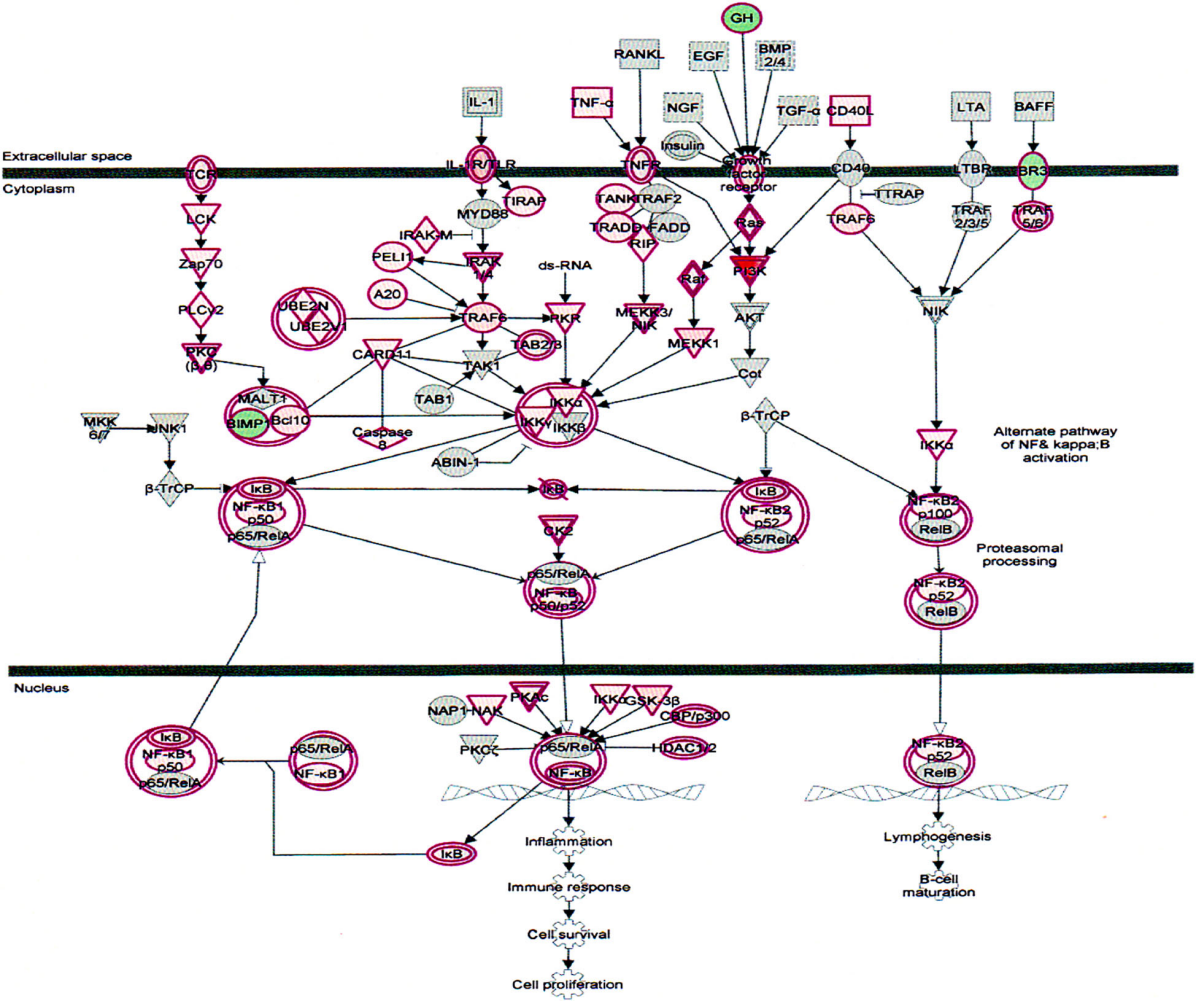
Effect on nuclear factor kappaB (NF-κB) in plasma of total mRNAs obtained from δ-tocotrienol treatment of hepatitis C patients. δ-Tocotrienol modulates NF-κB transcription factors, which are key regulators of gene expression and act in response to stress and the development of innate and acquired immunity. A number of NF-κB activators mediate the site-specific phosphorylation of serine on IκB (inhibitor of NF-κB), there by marking IκB for ubiquitination and subsequent proteasomal destruction

The nuclear factor kappa B (NF-κB) transcription factors are key regulators of gene expression and acts in response to stress and the development of innate and acquired immunity [30]. A multitude of extracellular stimuli (such as cytokines, infections, oxidative, DNA-damaging agents, UV light, osmotic shock) can lead to NF-κB activation. NF-κB activators mediate the site-specific phosphorylation of serine on IκB (inhibitor of NF-κB), resulting in IκB ubiquitination and subsequent proteasomal destruction [31]. The pathway highlights the important components of the NF-κB signaling pathway outlined in (Fig. 7). Inhibiting this pathway by proteasome inhibitors would possibly expected to cause cell death of infected hepatic cells.

**Fig. 8:**
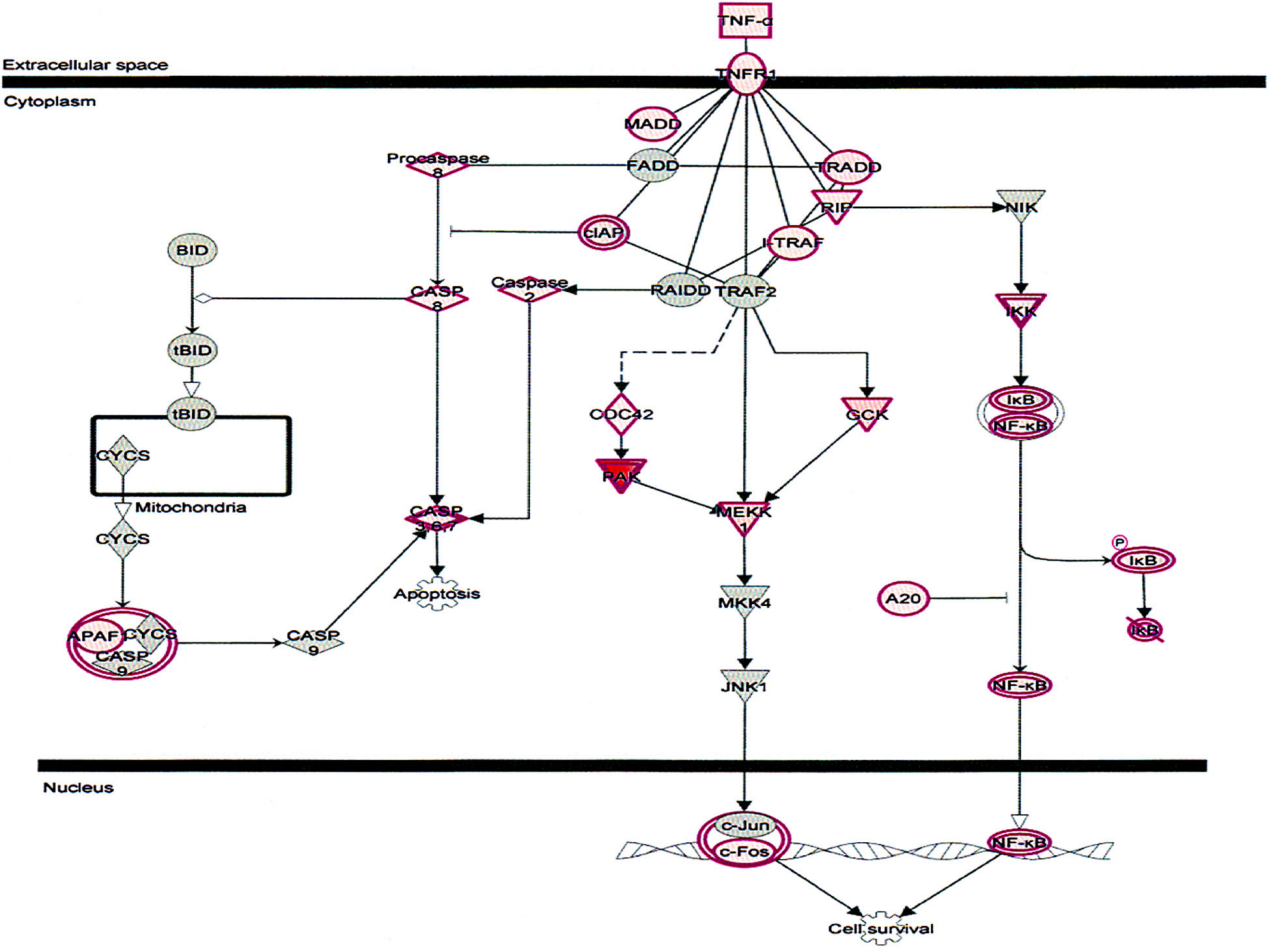
Effect on nitric oxide synthase (iNOS) in plasma of total mRNAs obtained from δ-tocotrienol treatment of hepatitis C patients. The iNOS was down-regulate by δ-tocotrienol treatment. It produces nitric oxide from L-arginine, a cytotoxic weapon generated by macrophages. The catalytic activity of iNOS is to kill or inhibit the growth of invading microorganisms. Nitric oxide is a free radical effector of the innate immune system that inhibits pathogen replication. A variety of extracellular stimuli (components of bacteria and fungi) can activate signaling pathways that help to initiate expression of iNOS

The catalytic activity of iNOS is to kill or inhibit the growth of invading viruses and microorganisms. It produces nitric oxide from L-arginine [32, 33]. Nitric oxide is a free radical effector of the innate immune system that can directly inhibit pathogen replication. A variety of extracellular stimuli can activate signaling pathways that converge to initiate expression of iNOS. Moreover, components of cell wall of bacteria (lipopolysaccharide; LPS) or fungi trigger the innate immune signaling cascade leading to expression of iNOS [34–36]. This leads to activation of NF-κB and p38 MAPK signaling pathways [37]. NF-κB in the nucleus binds to NF-κB elements in the iNOS 5′ flanking region, triggering iNOS transcription. Cytokines released from the infected host cell also activate nitric oxide production. IFNγ activates JAK family kinases to trigger JAK/STAT signaling, leading to synthesis of the transcription factor IRF1 and stimulation of a large number of iNOS mRNA transcription [38]. The iNOS signaling pathways (Fig. 8) shows all possible regulators of production of nitric oxide, and highlights the important molecular events leads to production in macrophages. Collectively, IFN-γ induced by δ-tocotrienols would be expected to modulate the JAK/STAT pathway and NO production.

**Fig. 9:**
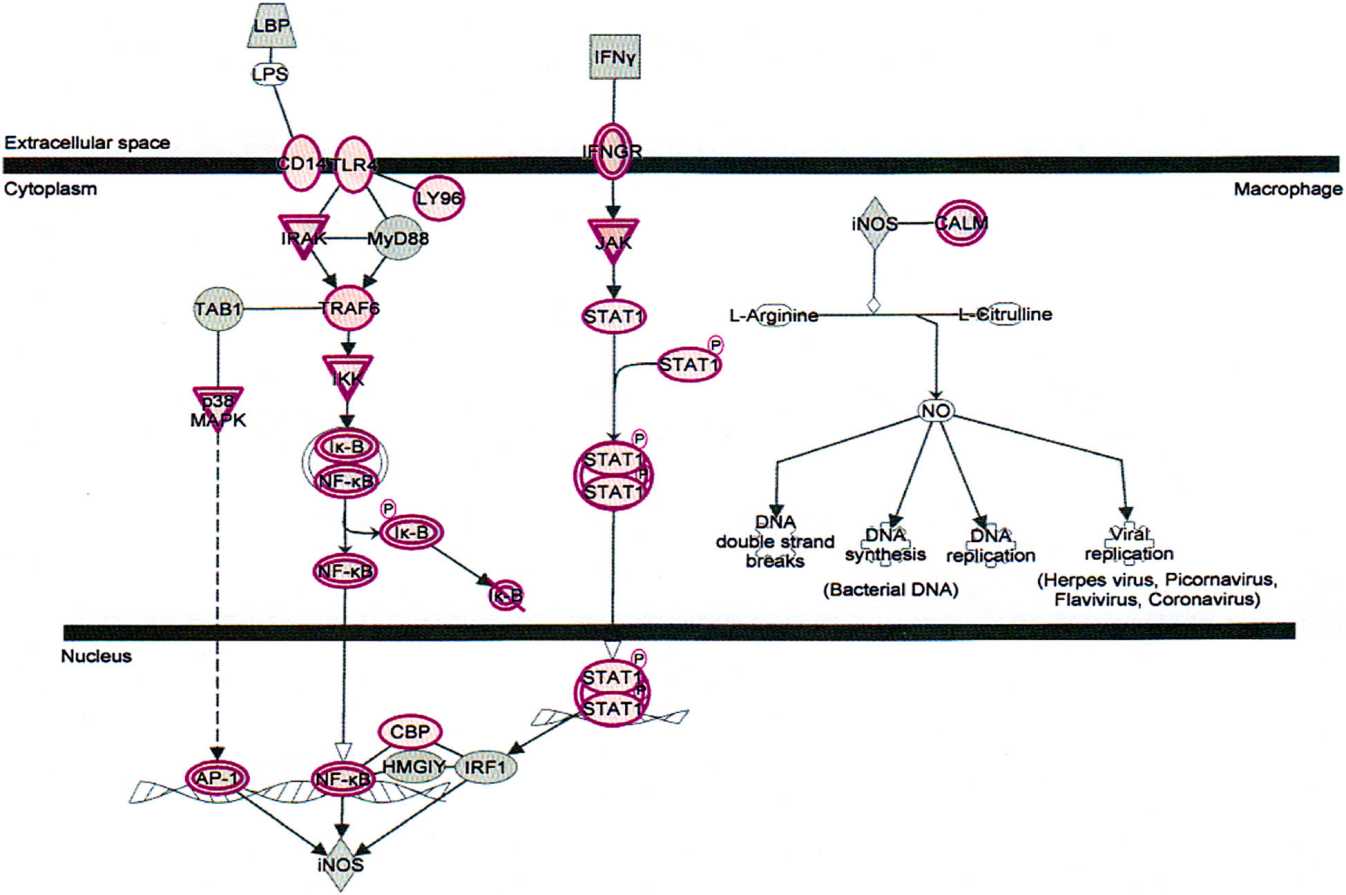
Effect on interleukin-6 (IL-6) regulator of gene expression in plasma of total mRNAs obtained from δ-tocotrienol treatment of hepatitis C patients. The IL-6 was down-regulated by δ-tocotrienol treatment, and is considered a regulator of acute phase responses and a lymphocyte stimulatory factor. The most important role of IL-6 is for the management of infection and inflammatory diseases. The transcription of IL-6 gene is stimulated by TNF-α and IL-1 via activation of NF-κB

Interleukin-6 (IL-6) is a regulator of acute phase responses and a lymphocyte stimulatory factor. The central role of IL-6 is for the management of infectious and inflammatory diseases [39]. IL-6 responses transmitted through glycoprotein 130 (GP130), which serves as the universal signal-transducing receptor subunit for all IL-6 related cytokines. Moreover, IL-6-type cytokines utilize tyrosine kinases of the Janus kinase (JAK) family and signal transducer/activators of STAT transcription family as major mediators of signal transduction [40]. In addition to the JAK/STAT pathway of signal transduction, IL-6 also activates the extracellular signal-regulated kinases (ERK1/2) of the mitogen activated protein kinase (MAPK) pathway (Fig. 9). The upstream regulators of ERK1/2 include RAS and the src homology-2 containing proteins GRB2 and SHC. The SCH protein activate by JAK2 and thus serves as a link between the IL-6 activated JAK/STAT and RAS-MAPK pathways shown in IL-6 signaling pathway Fig. 9 [41]. Furthermore, phosphorylation of MAPKs in response to IL-6 activated RAS results in the activation of nuclear factor IL-6 (NF-IL-6), which in turn stimulates the transcription of the IL-6 gene. IL-6 gene transcription is also stimulated by TNF-α and IL-1 via activation of NF-κB [41–43]. The tumor necrosis factor receptor (TNFR1) belongs to a family of 20 in mammalian cells.

**Fig. 10:**
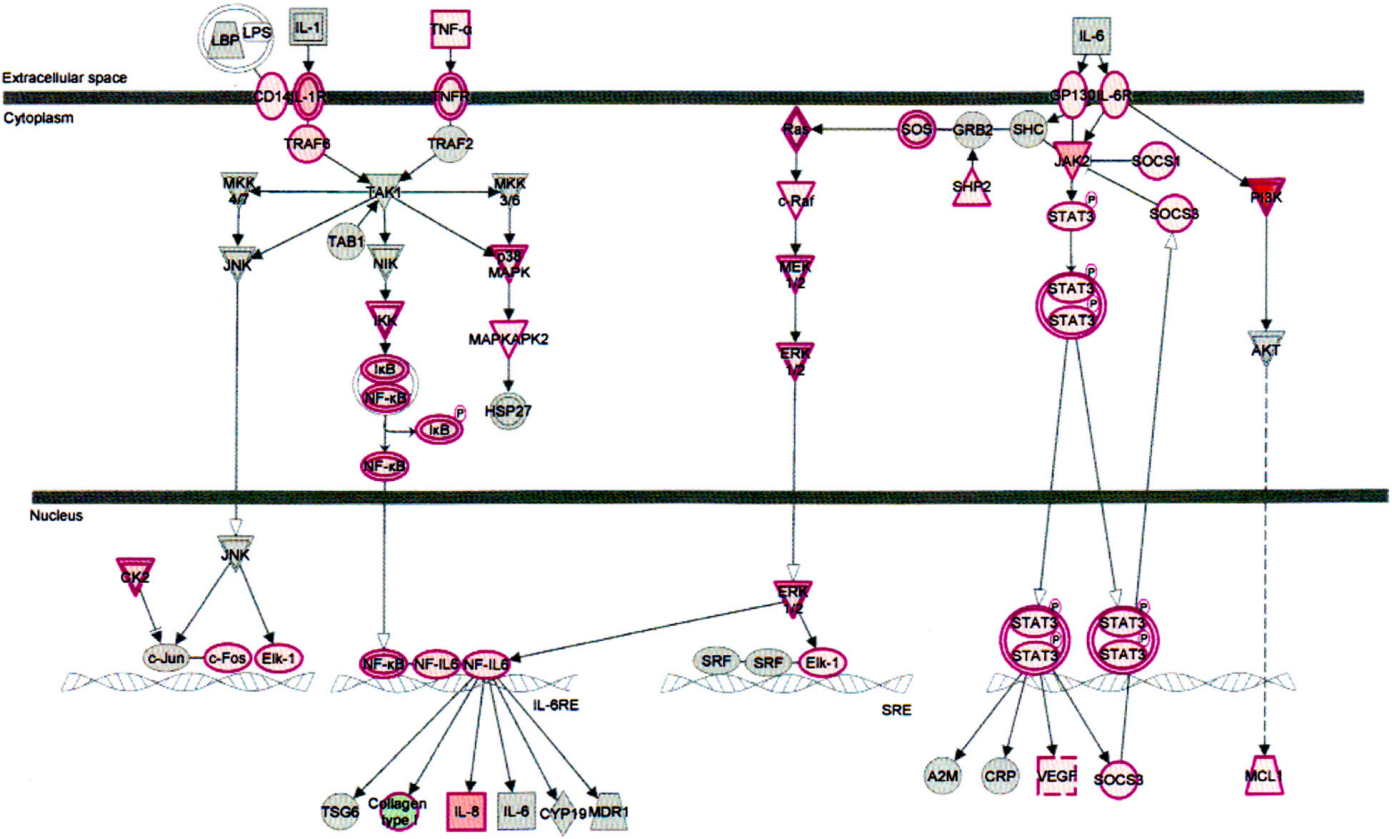
Effect on tumor necrosis factor receptor1 (TNFR1) regulator of gene expression in plasma of total mRNAs obtained from δ-tocotrienol treatment of hepatitis C patients. The TNFR1 was down-regulated by δ-tocotrienol treatment, and belongs to a family of 20 in mammalian cells. TNF-α is an important cytokine involved in cell proliferation, differentiation, apoptosis, modulates immune responses and induction of inflammation. TNF-α functions through two receptors, TNFR1 and TNFR2. TNFR1 is expressed in human tissue, and TNFR2 is expressed in immune cells

TNF-α, an important cytokine involves in cell proliferation, differentiation, and apoptosis modulate immune responses and induction of inflammation [44]. TNF-α functions through two receptors, TNFR1 TNFR2. TNFR1 is expressed in human tissue and TNFR2 expressed in immune cells (Fig. 10) [44, 45]. δ-Tocotrienol also inhibits expression of IL-6 and TNFR induction in chronic hepatitis C patients.

**Fig. 11:**
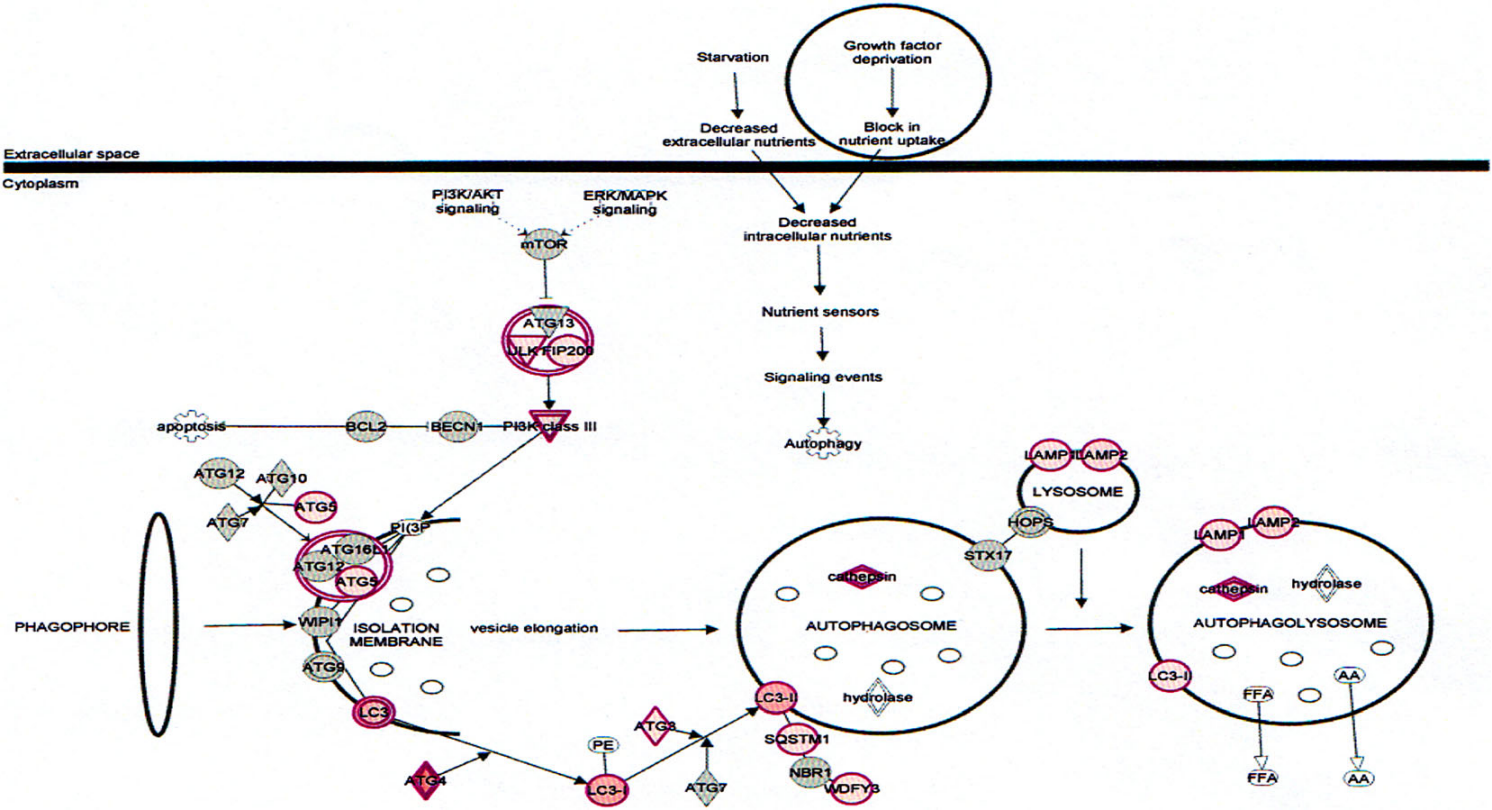
Effect on autophagy in plasma of total mRNAs obtained from δ-tocotrienol treatment of hepatitis C patients. The autophagy modulated by δ-tocotrienol treatment of hepatitis C patients:. Autophagy is a general term for the basic catabolic mechanism that involves cellular degradation of unnecessary or dysfunctional cellular components through the actions of lysosome. Autophagy is generally activated by conditions of nutrient deprivation but it has also been associated with physiological as well as pathological processes such as development, differentiation, neurodegenerative diseases, stress, infection, and cancer. The mammalian target of rapamycin (mTOR) kinase is a critical regulator of autophagy induction

Autophagy is a basic catabolic mechanism that involves cellular degradation of unnecessary or dysfunctional cellular components through the actions of liposome [46, 47]. Autophagy is generally activate by condition of nutrient deprivation but has also been associated with physiological as well as pathological processes such as development, differentiation, neurodegenerative diseases, stress, infection, and cancer [47–49]. The mammalian target of rapamycin (mTOR) kinase is a critical regulator of autophagy induction, with activated mTOR (AKT and MAPK signaling) suppressing autophagy, and negative regulation of mTOR (AMPK and p53 signaling) promoting it [48]. The autophagy pathway (Fig. 11) highlights the key molecular events involved in triggering autophagy. Inhibiting the proteasome activity also causes the onset of autophagy, as observed with δ-tocotrienol treatment.

**Fig. 12:**
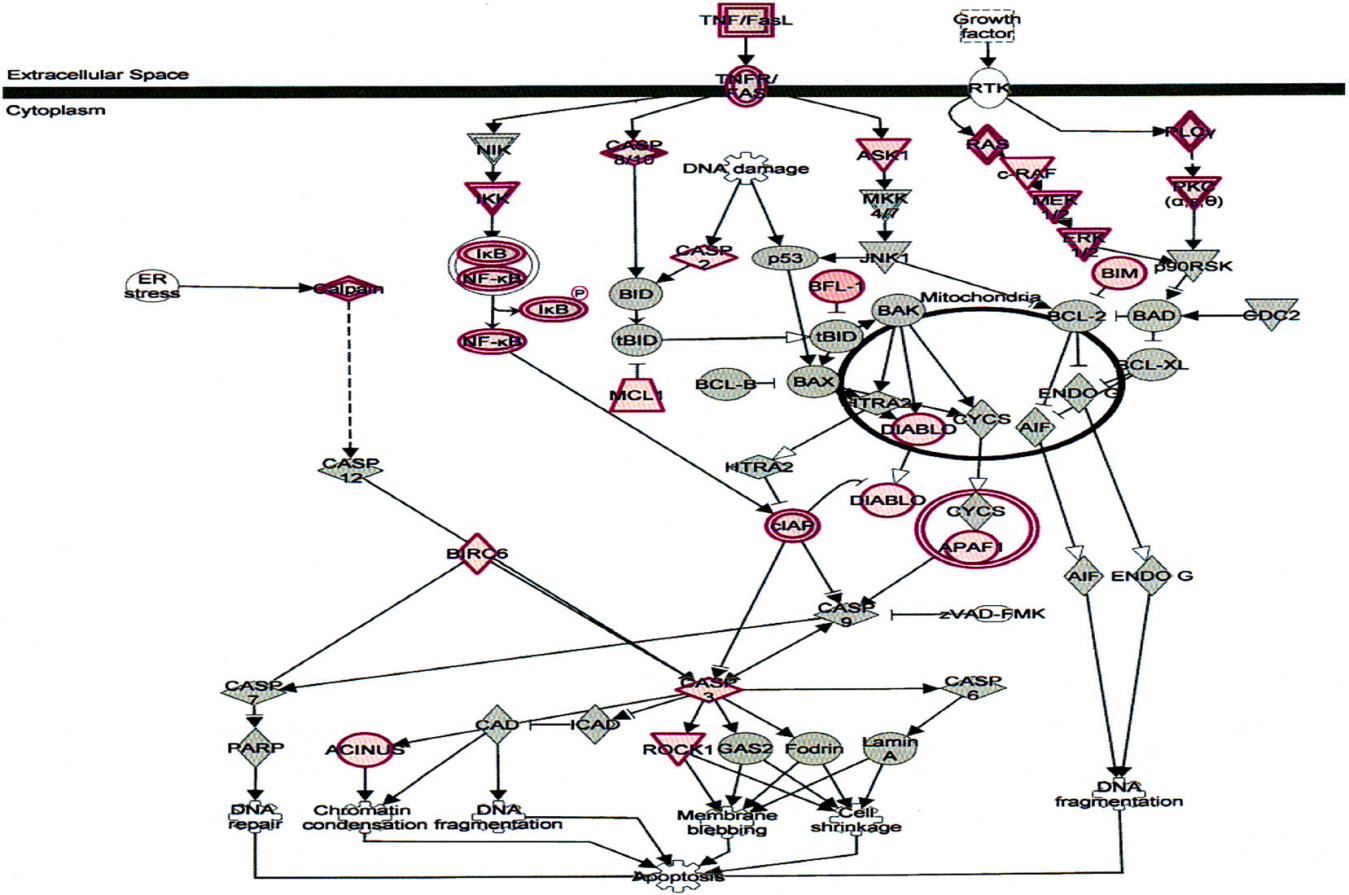
Effect on apoptosis in plasma of total mRNAs obtained from δ-tocotrienol treatment of hepatitis C patients. Apoptosis modulated by δ-tocotrienol treatment of hepatitis C patients. Apoptosis is a coordinated energy-dependent process that involves the activation of a group of cysteine proteases called caspases and a cascade of events that link the initiating stimuli to programmed cell death. There are two main pathways of apoptosis, the intrinsic and extrinsic as shown here

Whereas, apoptosis is a coordinated energy-dependent process that involves the activation of a group of cysteine proteases called caspases and a cascade of events that link the initiating stimuli to programmed cell death [50]. The two main pathways of apoptosis are the intrinsic and extrinsic pathways. Each pathway requires specific triggers to initiate a cascade of molecular events that converge at the stage of caspase-3 activation [50]. The activation of caspase-3 in turn triggers an execution pathway resulting in characteristic cytomorphological features including cell shrinkage, membrane blabbing, chromatin condensation and DNA fragmentation [51]. Further details of intrinsic and extrinsic pathways were found in the attached Ingenuity Apoptosis Signaling Pathway (Fig. 12), which highlights the key molecular events involved in trigging apoptosis.

Beside these, other regulators were also affected by δ-tocotrienol treatment of hepatitis C patients, and they are interferon signaling, IL-2 signaling, and HMGB1 signaling, Cardiac hypertrophy signaling, Th1 and Th2 activation pathway, production of nitric oxide and reactive oxygen species in macrophages, Osteoarthritis pathway, PPAR signaling, type,I diabetes mellitus signaling, Type II diabetes mellitus, and insulin receptor signaling. In summary, EIF2 signaling regulator is at the top of the canonical pathway list but its fold change expression value is 221 as compared to protein ubiquitination pathway is 265 fold. On the other hand, osteoarthritis (210 fold), mammalian target of rapamycin (mTOR-201 fold), IL-8 (197 fold), Th1-Th2 (185 fold), PPARα/RXRα activation (180 fold), NF-κB (181 fold), IL-6 (128 fold), Type II diabetes mellitus signaling (128 fold), and nitric oxide signaling in cardiovascular system (113 fold), all have lower fold change expression compared to EIF2. This indicates the importance of δ-tocotrienol on so many biological activities and signaling pathways (Table 11). The importance of most of these regulators was discussed in our several publications during course of the last decade [1, 11–15].

## Conclusions

Present results of fold-change expression data analyzed by “Ingenuity Pathway Analysis” describe the effect of δ-tocotrienol in chronic hepatitis C patients on biological mechanisms at molecular level. It also revealed an insight of correlation of signaling pathways and transcriptional factors. Recently, two comprehensive reviews on the several biological activities of tocotrienols as hypocholesterolemic, anti-inflammatory, anticancer, antioxidant, neuroprotective, skin protection benefits, bone health and longevity have been published [52, 53]. These articles also cover the beneficial properties of different isomers of tocotrienols treatment along with possible mechanisms, signaling pathways in breast, prostate, pancreas, rectal cancers in cell lines and humans [52, 53]. Major signaling pathways that were affected by δ-tocotrienol treatment in chronic hepatitis C subjects are summarized in the Table 12. The collective results indicate that tocotrienols inhibit cancer cell proliferation, promotes cell cycle arrest, decreases angiogenesis and acts via multiple signaling pathways [1]. Our present results are consistent with these conclusions and δ-tocotrienol treatment of hepatitis C patients, acts by increasing cell death, and necrosis of malignant tumors, and by decreasing viral infection, cellular growth and proliferation, decreasing endocrine system disorders such as diabetes mellitus, and mobilization of calcium. Therefore, tocotrienols can safely be used for hepatitis C patients, without any side effects.

**Table 12:** Major signaling pathways affected by δ-tocotrienol treatment in chronic hepatitis C subjects

Down-regulated by δ -tocotrienol treatment	Up-regulated by δ-tocotrienol treatment
Proliferation of immune cells	Cell death and survival
Proliferation of mononuclear leukocytes	Necrosis of malignant tumor
Viral infection	Gene expression
Free radical scavenging	Organismal Death
Endocrine system disorder, Diabetes mellitus	Cell death of cancer cells
Mobilization of Ca2+	Cell death of tumors
Replication of virus	
HIV infection, replication of Influenza virus	

## Additional files

Additional file 1: **Table S1.** Effect of d-tocotrienol on down-regulation of gene expression of "Molecules" (1-75) of IPA analyses in hepatitis C patients. (XLS 68 kb)

Additional file 2: **Table S2.** Effect of d-tocotrienol on down-regulation of gene expression of "Molecules" (76-150) of IPA analyses in hepatitis C patients. (XLS 68 kb)

Additional file 3: **Table S3.** Effect of d-tocotrienol on down-regulation of gene expression of "Molecules" (151-220) of IPA analyses in hepatitis C patients. (XLS 67 kb)

## Abbreviations

EIF2: Eukaryotic translation initiation factors
ICAM1: Intercellular adhesion molecule1
IL-6: Interleukin-6
IPA: Ingenuity Pathway Analysis
mTOR: Mammalian target of rapamycin
NF-κB: Nuclear factor kappaB
TNF-α: Τumor necrosis factor-α
VCAM1: Vascular cell adhesion molecule1

## References

[1] Qureshi AA, Eleanor Z, khan DA, Shahida M, Silswal N, Qureshi N (2018). Proteasomes inhibitors modulate anticancer and anti-proliferative properties via NF-κB signaling, and ubiquitin-proteasome pathways in cancer cell lines of different organs. Lipids Health Dis.

[2] Shepard CW, Finelli L, Alter MJ (2005). Global epidemiology of hepatitis C virus infection. Lancet Infect Dis.

[3] Hamid S, Umar M, alam A, Siddiqui A, Qureshi H, Butt J (2004). PSG consensus statement on management of hepatitis C virus infection-2003. J Pak Med Assoc.

[4] DeRisi J, Penland L, Brown PO, Bittner ML, Meltzer PS, Ray M, Chen Y, Su YA, Trent JM (1996). Use of a cDNA microarray to analyse patients in human cancer. Nat Genet.

[5] Patil MA, Chua MS, Pan KH, Lin R, Leh, Cheung ST, Ho C, Li R, Fan ST, Cohen SN, Chen X, So S (2005). An integrated data analysis approach to characterize genes highly expressed in hepatocellular carcinoma. Oncogene.

[6] Shackel NA, McGuinness PH, Abbott CA, Correll MD, McCaughan GW (2002). Insight into the pathobiology of hepatitis C virus associated cirrhosis: analysis of intrahepatic differential gene expression. Am J Pathol.

[7] Smith MW, Yue ZN, Korth MJ, Do HJ, Boix L, Fausto N, Bruix J, Carithers RL, Katze MG (2003). Hepatitis C virus and liver disease: global transcriptional profiling and identification of potential markers. Hepatology (Baltomore, MD).

[8] Zein NN (2000). Clinical significance of hepatitis C virus genotypes. Clin Microbiol Rev.

[9] Galardi S, Fatica A, Bachi A, Scaloni A, Presutti C, Bozzoni I (2002). Purified box C/D snoRNAs are able to reproduce site-specific 2;-O-methylation of target RNA in vitro. Mol Cell Biol.

[10] Tycowski KT, Shu MD, Steitz JA (1993). A small molecular RNA is processed from an intron of the human gene encoding ribosomal protein S3. Genes Dev.

[11] Albig W, Kioschis P, Poustka A, Meergans K, Doeneck D (1997). Human histone gene organization: nonregular arrangement within a large cluster. Genomics.

[12] Zhang DD, Wang WT, Xiong J, Xie XM, Cui SS, Zhao ZG, Li MJ, Zhang ZQ, Hao DL, Zhao X, Li J, Wang J, Chen HZ, Lv X, Liu DP (2017). Long noncoding RNA LINC00305 promotes inflammation by activating the AHRR-NF-κB pathway in human monocytes. Sci Rep.

[13] Doyard M, Fatih N, Monnier A, Island ML, Aubry M, Leroyen P, Bouvet R, Charles G, Loreal O, Guggenbuhl P (2012). Iron excess limits HHIPL-2 gene expression and decreases osteoblastic activity in human MG-63 cells. Osteoporos Int.

[14] Strichman-Almashanu L, Bustin M, Landsman D (2003). Retroposed copies of the HMG genes: a window to genome dynamics. Genome Res.

[15] Rogalla P, Botda Z, Meyer-Bolte K, Tran KH, Hauke S, Nimzyk R, Bullerdiek J (1998). Mapping and molecular characterization of five HMG1-related DNA sequences. Cytogen Cell Genet.

[16] Kiss T (2002). Small nuclear RNAs: an abundant group of noncoding RNAs with diverse cellular functions. Cell.

[17] Stove EH, Konstantinopoulos PA, Matulonis UA, Swisher EM (2016). Biomarkers of response and resistance to DNA repair targeted therapies. Clin Cancer Res.

[18] Arno G, Carss KJ, Hull S, Zihni C, Robson AG, Fiorentino A, Hardcastle AJ, Holder GE, Cheetham ME, Plagnol V, Moore AT, Raymond FL, Matter K, Balda MS, Webster AR, UK Inherited Retinal Disease Consortium, NIHR Bioresource-Rare Disease Consortium (2017). Biallelic mutation of ARHGEF18, involved in the determination of epithelial apicobasal polarity, causes adult-onset retinal degeration. Am J Hum Genet.

[19] Houchins JP, Yabe T, McSherry C, Bach FH (1991). DNA sequence analyses of NKG2, a family of related cDNA clones encoding type II integral membrane proteins on human natural killer cells. J Exp Med.

[20] Albig W, Doenecke D (1997). The human histone gene cluster at the D6S105 locus. Human Genet.

[21] Marzluff WF, Gongidi P, Woods KR, Jin J, Maltais LJ (2002). The human and mouse replication-dependent histone genes. Genomics.

[22] Dayan A, Bertrand R, Beachemin M, Chahla D, Mamo A, Filion M, Skup D, Massie B, Jolivet J (1995). Cloning and characterization of the human 5,10-methenyltetrahdrofolate synthase-encoding cDNA. J Gene.

[23] Bertrand R, Beauchemin M, Dayan A, Quimet M, Jolivet J (1995). Identification and characterization of human mitochondrial methenyltetrahdrofolate synthetase activity. Biochem Biophys Acta.

[24] Murry JL, Sheng J, Rubin DH (2014). A role for H/ACA and C/D small nucleolar RNAs in viral replication. Mol Biotechnol.

[25] Zhang D, Zhang G, Hayden MS, Greenbaltt MB, Bussey C, Flavell RA, Ghosh S (2004). A toll-like receptor that prevents infection by urophathogenic bacteria. Science.

[26] Kien E, Means TK, Heine H (2000). Toll-like receptor 4 imparts lagand-specific recognition of bacterial lipopolysaccharide. J Clin Invest.

[27] Silva CM (2004). Role of STATs as downstream signal transducers in Src family kinase-mediated tumorigenesis. Oncogene.

[28] Lin CP, Cao X (2006). Structure, function, and regulation of STAT protein. Mol BioSyst.

[29] Yuan ZL, Guan YJ, Wei W, Wang L, Kane AB, Chin YE (2004). Central role of the threonine residue within the p+1 loop of trceptor tyrosine kinase in STAT3 constitutive phosphorylation in metastatic cancer cells. Mol Cell Biol.

[30] Karin M (1999). The beginning of the end: IκB kinase (IKK) and NF-κB activation. J Bol Chem.

[31] Palombella VJ, Rando OJ, Goldberg AL, Maniatis T (1994). The ubiquitin-proteasome pathway is required for processing the NF-κB precursor protein and activation of NF-κB. Cell.

[32] Moncada S, Higgs A (1993). The L-arginine-nitric oxide pathway. N Engl J Med.

[33] Moncada S, Palmer RM, Higgs EA (1991). Nitric oxide: physiology, pathophysiology and pharmacology. Pharmacol Rev.

[34] Forstemann U, Closs EI, Pollock JS, Nakane M, Schwarz P, Gath I, Kleinert H (1994). Nitric oxide synthase isozyme: characterization, purification, molecular cloning and functions. Hypertension.

[35] Nadaud S, Sobrier F (1996). Molecular biology and molecular genetics of nitric oxide synthase genes. Clin Exp Hypertens.

[36] Nathan C, Xie O (1994). Nitric oxide synthase: roles, tolls and controls. Cell.

[37] Qureshi N, Vogel SN, Van Way C, Papasian CJ, Qureshi AA, Morrison DC (2005). The proteasome. A central regulator of inflammation and macrophage function. Immunol Res.

[38] Ma C, Wang DL, Li M, Cai W (2015). Anti-inflammatory effect of resveratrol through the suppression of NF-kB and JAK/STAT signaling pathway. Acta Biochim Biophys Sin.

[39] Kallen KJ, zum Buschenfelde KH, Rose-John S (1997). The therapeutic potential of interleukin-6 hyperagonists and antagonists. Expert Opin Investig Drugs.

[40] Heinrich PC, Behrmann I, Muller-newen G, Schaper F, Graeve F (1998). Interleukin-6-type cytokine signaling through the gp 130/Jak/STAT pathway. Biochem J.

[41] Brandt C, Pedersen BK. The role of exercise-induced myokines in muscle homeostasis and the defense against chronic diseases. J Biomed Biotechnol 2010; Article ID 520258, 6 pages. Doi:10.1155/2010/520258. 10.1155/2010/520258 PMC2836182 20224659

[42] Munoz-Canoves P, Scheele C, Pedersen BK, Serrano AL (2013). Interkin-6 myokine signaling in skeletal muscle: a double-edged sword?. FEBS J.

[43] Meador BM, Krzyszton CP, Johnson RW, Huey KA (2008). Effects of IL-10, and age on IL-6, IL-1b, and TNF-α responses in mouse skeletal and cardiac muscle to an acute inflammatory insult. J Appl Physiol.

[44] Beutler B, Greenwald D, Hulmes JD, Chan M, Pan YC, Matuison J, Ulevith R, Cerami A (1985). Identity of tumor necrosis factor and macrophage-secreted factor cachectin. Nature.

[45] Soranzo C, Perego P, Zunino F (1990). Effect of tumor necrosis factor on human tumor cell lines sensitive and resistant to cytotoxic drugs, and its interaction with chemotherapeutic agents. Anti-Cancer Drugs.

[46] Ziparo E, Petrungaro S, Marini ES, Starace D, Conti S, Facchiano A, Filippini A, Giampietri C (2013). Autophagy in prostate cancer and androgen suppressioin therapy. Int J Mol Sci.

[47] Rubinsztein DC, Bento CF, Deretic V (2015). Therapeutic targeting of autophagy in neurodegenerative and infectious diseases. J Exp Med.

[48] Nedelsky NB, Todd PK, Taylor JP (2008). Autophagy and ubiquitin-proteasome system: collaborators in neuroprotection. Biochim Biophys Acta.

[49] Zhu K, Dunner K, McConkey DJ (2010). Proteasome inhibitors activate autophagy as a cytoprotective response in human prostate cancer cells. Oncogene.

[50] King LB, Ashwell JD (1994). Thymocyte and T cell apoptosis: is all death created equal?. Thymus.

[51] Zhang N, Hartig H, Dzhagalov I, Draper D, He YW (2005). The role of apoptosis in the development and function of T lymphocytes. Cell Res.

[52] Kanchi MM, Shanmugan MK, Rane G, Sethi G, Kumar AP (2017). Tocotrienols: the unsaturated sidekick shifting new paradigms in vitamin E therapeutics. Drug Discov Today.

[53] Sailo BL, Banik K, Padmavathi G, Javadi M, Bordoloi D, Kunnumakkara AB (2018). Tocotrienols: the promising analogue of vitamin E for cancer therapeutics. Pharmacol Res.

[54] Qureshi AA, Khan DA, Mahjabeen W, Trias AM, Silswal N, Qureshi N (2015). Impact of δ-tocotrienol on inflammatory biomarkers and oxidative stress in hypercholesterolemic subjects. J Clin Exp Cardiolog.

